# Sequential Nitrile Amidination–Reduction as a Straightforward Procedure to Selective Linear Polyamine Preparation

**DOI:** 10.1021/acs.joc.3c02128

**Published:** 2023-11-25

**Authors:** Antonio Peñas-Sanjuán, Jose J. Chica-Armenteros, Rubén Cruz-Sánchez, Celeste García-Gallarín, Manuel Melguizo

**Affiliations:** Departamento de Química Inorgánica y Orgánica. Facultad de Ciencias Experimentales, Universidad de Jaén, 23071 Jaén, Spain

## Abstract

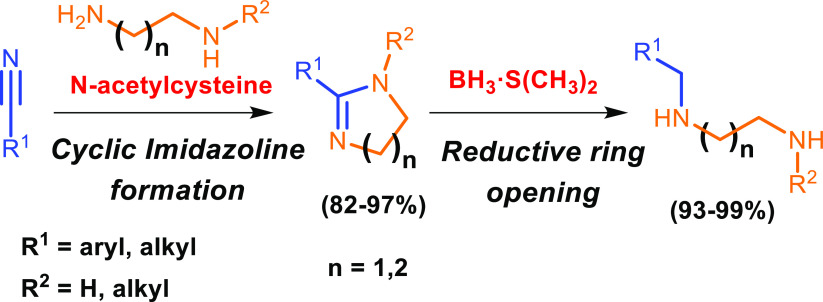

A straightforward strategy toward the efficient synthesis of linear saturated polyamines containing 1,2-diaminoethane and/or 1,3-diaminopropane fragments has been developed. The procedure is based on the chemistry of 5- and 6-membered cyclic amidines, including their efficient synthesis from nitrile precursors and subsequent chemoselective reductive-opening by a borane–dimethyl sulfide complex. This two-step procedure provides a robust methodology for the synthesis of linear polyamine skeletons under nonharsh conditions and free of using selective protective groups or tedious workups.

## Introduction

Low molecular weight saturated polyamines are ubiquitous metabolic substances found in all eukaryotic cells. The best-known members of such naturally occurring polyamines are likely the diamine putrescine (1,4-diaminobutane), together with the triamine spermidine and tetraamine spermine. Various relevant biological roles have been attributed to these linear saturated polyamines (LSPs), such as key agents in the division of eukaryotic cells or in the synthesis of proteins and nucleic acids,^1−4^ which make polyamine synthesis a field of interest regarding their potential biological and pharmacological roles as antineoplastics in cancer therapy,^5,6^ as agents against neurodegenerative disorders (Alzheimer’s and Parkinson’s diseases or amyotrophic lateral sclerosis),^7−9^ or as antimicrobial drugs.^10^

In addition to these biological roles, but in direct relation with them, one of the most fruitful lines of the nonviral transfection technologies has pivoted around the essential task that saturated linear polyamines play in the stabilization and delivery of RNAs.^11−14^ Such short, linear polyamines, which are mostly made by the linear combination of a small number (1 to 5) of ethylenimine and/or propyleneimine units, can provide effective antiviral treatments, including that for the recent SARS-CoV.^15^

These and other technical applications (ion scavengers,^16^ self-healing polymers,^17,18^ molecular sensors,^19^ among others) of linear, short-chain polyamines make these structures interesting targets from a synthetic point of view. However, despite the apparent structural simplicity of these molecules, their syntheses remain far from easy. Thus, although in a first approach, the nucleophilic character of the amino groups could be thought as exploitable to extend the chain of a precursor amino group, the capacity of the primary amino group to suffer double alkylation, so affording branched amines, imposes extensive protection–deprotection strategies to achieve the pursued LSP molecules free from branched polyamine secondary products. Besides, polyamines are not easy to purify by conventional normal-phase chromatography on silica gel due to their pronounced basicity. Consequently, preparation of polyamines, at the level of grams or larger quantities, through conventional methodologies, including Michael additions to α, β-unsaturated nitriles, alkylations of amines and sulfonamides, or reductive alkylations and acylations, followed by reduction of the thus-obtained amides or azides,^20^ normally requires complex procedures that seriously hinder the synthesis of LSPs, from the technical and economical points of view.^3,20^

Opposite to that general scenery, we present here an easy procedure capable of producing LSPs from a nitrile and a linear polyamine that includes a terminal fragment of 1,2-ethylenediamine or 1,3-propylenediamine. The procedure is performed in two steps: (a) transformation of the nitrile group into a 5- or 6-membered cyclic amidine (2-imidazolines and 3,4,5,6-tetrahydropyrimidines, respectively),^21^ followed by (b) reductive ring opening of the cyclic amidine to selectively yield a new linear polyamine that keeps intact the carbon skeleton of the starting nitrile, but extends it with the amine structure.^22,23^

Since the first synthesis of 2-methyl-imidazoline by heating *N*,*N*′-diacetylethylenediamine in dry hydrogen chloride, reported by Hofmann,^24^ many synthetic methodologies to obtain 2-imidazolines have been developed based on the reaction of 1,2-diamines, isocyanides, amidines, imines, amides, aziridines, and cyanides, which extensively entail the use of transition metals such as Cu, Ag, Pd, Ni, Rh, Ti, or W as catalysts.^25,26^ In contrast, we report the synthesis of 2-imidazolines and 3,4,5,6-tetrahydropyrimidines, from diamines and nitriles, by using *N*-acetylcysteine as an organocatalyst able to promote the cyclic amidine formation in the absence of transition metals or metal ions, with excellent yields. Thus, although the *N*-acetylcysteine was reported by Lange et al.^21^ as a catalyst to obtain noncyclic amidines [R–C(NH_2_)=NH], from nitriles and ammonia, there are no precedents about the application of this procedure to obtain 2-imidazolines or 3,4,5,6-tetrahydropyrimidines, which could be attributable to a wrong idea that 2-imidazoline and 3,4,5,6-tetrahydropyrimidine formation is restricted by the nitrile nitrogen atom removed as ammonia.

Regarding reductive opening of 2-imidazolines and 3,4,5,6-tetrahydropyrimidines unsubstituted at the nitrogen atom N(3), precedents are rather scarce in the literature, because typically 2-imidazoline reductions are performed after the N(3)-alkylation with alkyl iodides, which renders 1,2,3-trisubstituted dihydroimidazolium ions highly reactive under reductive opening conditions. Thus, reductive opening conditions, of 2-imidazolines unsubstituted at the nitrogen atom N(3), are limited to use LiAlH_4_/THF, LiAlH_4_/AlCl_3_/THF, DIBAL/xylene, and NaBH_3_CN/EtOH, which are defined by long reaction times, low chemoselectivities, moderate yielding conversions, and tedious processing.^27^ In the present work, we have investigated the reductive opening of various 2-imidazolines and 3,4,5,6-tetrahydropyrimidines by using the borane–dimethyl sulfide complex as a reductive agent. As a result, reductive openings were performed in high yield and excellent chemoselectivity toward the obtaining of LSPs, avoiding N(3)-alkylation.

Therefore, the proposed sequential nitrile amidination–reduction methodology provides an efficient linear polyamine synthesis, which allows repetitive and sequential elongations of 1,2-ethylenediamine or 1,3-propylenediamine fragments to be performed through the unusual idea of using 2-imidazolines and 3,4,5,6-tetrahydropyrimidines as intermediates. As a corollary, starting from benzonitrile as the precursor, this two-step procedure of amine extension can be considered a simple and efficient route to achieve selective *N*-benzyl monoprotection of one of the primary amino groups in di- or triamines.

## Results and Discussion

**Scheme 1:**
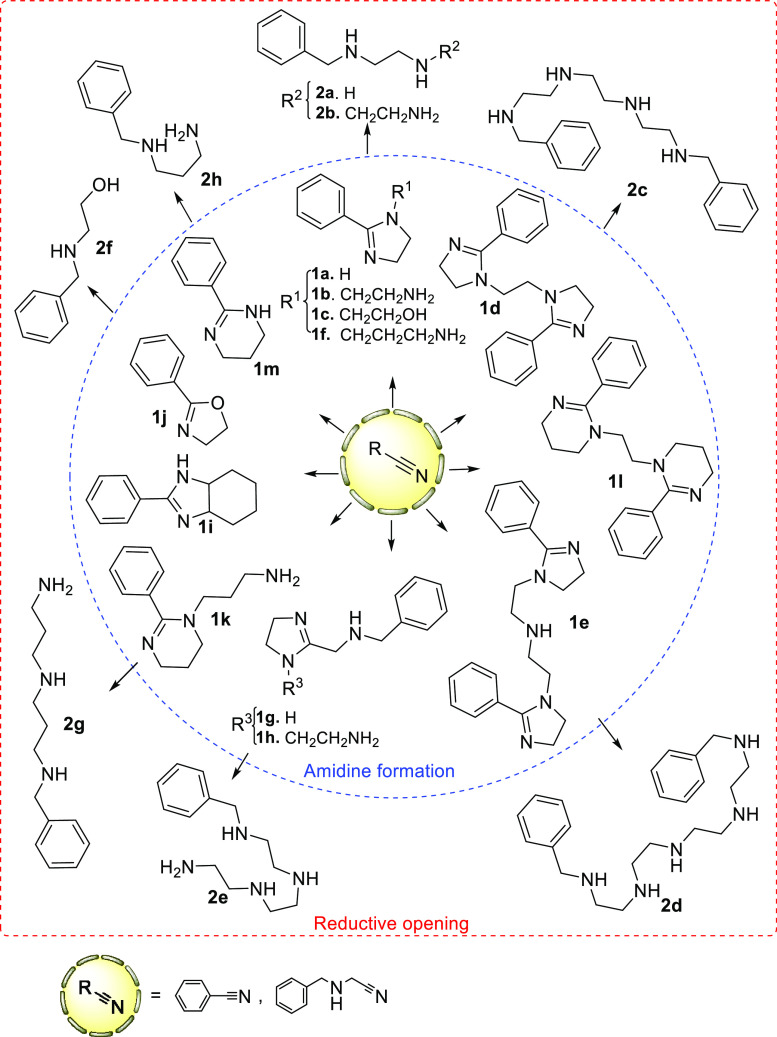
Synthesis of 5- and 6-Membered Cyclic Amidines and Subsequent Reductive Opening

In order to prove the synthetic transformation of nitriles into 5- or 6-membered cyclic amidines, under the organocatalytic influence of *N*-acetylcysteine, the reaction between benzonitrile and ethylenediamine was selected as a model reaction, which afforded an excellent yield of 2-phenyl-2-imidazoline (**1a**) in methanol under mild conditions (60 °C, 24 h). Conditions similar to these reported by Shäefer et al.^21^ for preparation of unsubstituted amidines (methanol, 1 equiv of *N*-acetylcysteine to 1 equiv of amine, 60 °C, 24 h) but modified to a lower excess of ethylenediamine (1.5 to 1 equiv of nitrile) afforded an excellent yield of 2-phenyl-2-imidazoline (**1a**). Other sulfide-based agents with potential catalytic activity for the transformation of nitriles into amidines, namely, Na_2_S and 2-mercaptoethanol, instead of *N*-acetylcysteine,^28^ were tested (MeOH, 60 °C up to 48 h) without success. Then, a large set of experiments using different 1,2-ethylenediamine or 1,3-diaminopropane fragments, as well as cyano groups, was performed under similar conditions in order to explore the scope of this first synthetic step (Scheme 1). For the preparation of double imidazolines, the excess was inverted (1.2 equiv of nitrile to 1 of amine) to ensure formation of double amidines as the sole product. In this step, the cyclic amidines (or oxazoline, **1j**) were obtained in good to excellent yields (Table 1). The only expected byproduct is ammonia, from the nitrile nitrogen atom, which will mostly escape from the reaction medium (MeOH, 60 °C) and get easily exhausted during workup.

**Table 1:**
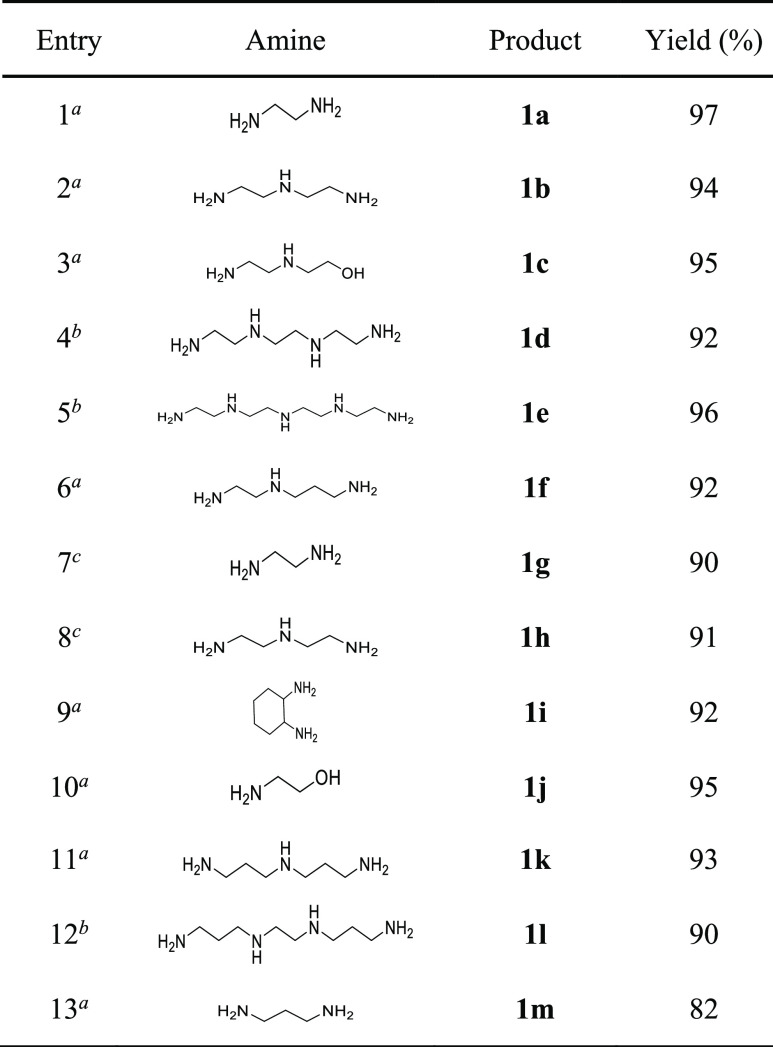
Synthesis of 5- and 6-Membered Cyclic Amidine Derivatives

a Benzonitrile (6.7 mmol), polyamine (10 mmol), and *N*-acetylcysteine (10 mmol) in MeOH (10 mL), at 60 °C, 24 h, Ar.

b Benzonitrile (24 mmol), polyamine (10 mmol), and *N*-acetylcysteine (10 mmol) in MeOH (10 mL), at 60 °C, 24 h, Ar.

c 2-(Benzylamino)acetonitrile (6.7 mmol), polyamine (10 mmol), and *N*-acetylcysteine (10 mmol) in MeOH (10 mL), at 60 °C, 24 h, Ar.

For the transformation of nitriles into cyclic amidines, the results reported in Scheme 1 and Table 1 show that 2-imidazolines and 3,4,5,6-tetrahydropyrimidines can be formed in good to excellent yields, noting the following aspects: (a) 2-imidazolines can be obtained from aryl or alkyl cyanides; (b) from the point of view of the reacting 1,2-diamines, 1,2-ethylenediamine, C-substituted 1,2-ethylenediamine (as 1,2-diaminocyclohexane), mono *N*-substituted 1,2-ethylene diamine, or linear unsubstituted polyethyleneimines are useful reactants to perform the transformation; (c) additionally, 2-imidazolines were formed at both ends of the polyethyleneimine chain from the tetra-amine or a longer oligomer; (d) monounsaturated heterocycles different to 2-imidazolines are formed, when appropriate amines react with nitriles, for example, 2-oxazolines or 3,4,5,6-tetrahydropyrimidines, when 2-aminoethanol or 1,3-diaminopropane derivatives, respectively, are reacted; (e) formation of 2-imidazolines are entirely favored over 3,4,5,6-tetrahydropyrimidines when both structures could be competitively obtained, which is in accordance with the higher thermodynamic stability of 2-imidazolines (entry 6, Table 1); and (f) as far as the cyclic amidine (or oxazoline) requires reaction of the nitrile carbon atom with two nucleophiles, high specificity is observed toward obtaining intramolecular (cyclic) products with respect to intermolecular, open-chain amidine products (not detected).

**Scheme 2:**
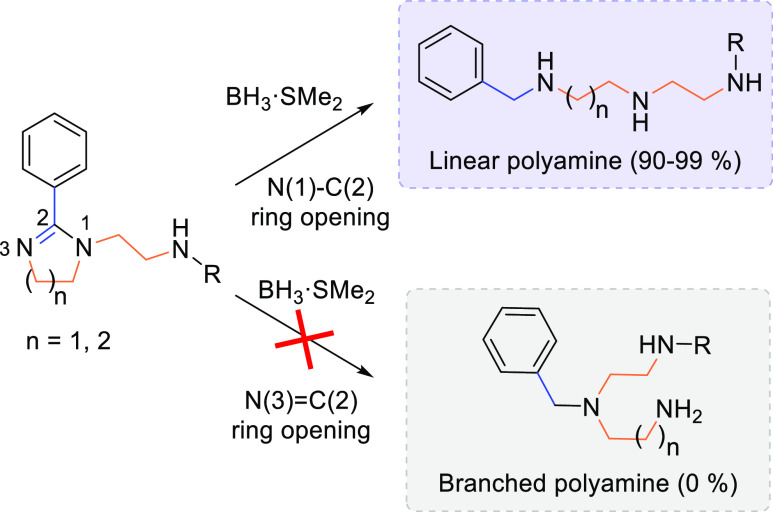
Chemoselectivity of Borane as Reductive Agent of Cyclic Amidines

Regarding reductive ring openings, the selection of an agent to accomplish this transformation was directed toward transition metal-free reagents to avoid potential generation of chelate complexes with the 1,2- or 1,3-diamine resulting fragments, that could complicate purification of the products. Then, borane, whose ability to reduce amides, lactams, and nitriles to amines is well-documented,^29^ was the reagent of choice. Furthermore, the high basicity of amidines would match with the Lewis acid character of borane, favoring the coordination of the reagent at the reaction site. Among the commercially available borane complexes, that of dimethyl sulfide ensures displacement by amidine and amine functions owing to the better electron pair donation ability of these nitrogen functions. Thus, the borane–dimethyl sulfide complex, as a reductive agent, showed specific production of linear polyamines, yielding exclusively nonbranched polyamines from N(1)-substituted-2-imidazoline and N(1)-substituted-3,4,5,6-tetrahydropyrimidines (see Table 2), which reveals the great chemoselectivity of borane as a imidazoline reductive agent (Scheme 2). The reduction outcome is linear polyamines based on sequences of ethylenediamine and/or propylenediamine units. In the case of benzonitrile, the resulting linear amines can be regarded as *N*-benzyl protected at one or two of their terminal amino groups. From this point of view, the sequential amidination–reduction applied on benzonitrile constitutes an effective procedure for the selective terminal *N*-benzyl monoprotection of di- and triamines possessing a fragment of 1,2-ethylenediamine or 1,3-propylenediamine (Table 2, entries 1,2,5–8).

**Table 2:**
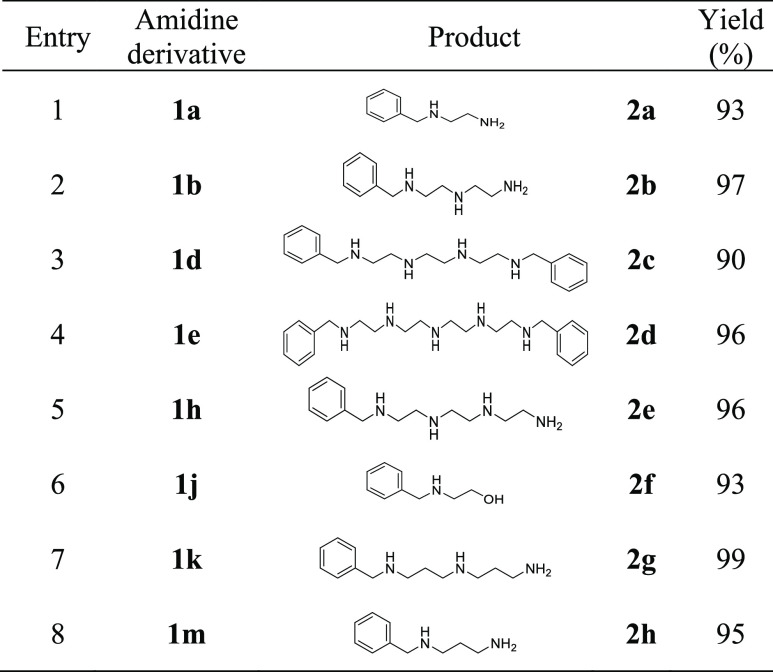
Reductive Opening of Cyclic Amidine Derivatives^a^

a Imidazoline (10 mmol) and borane–dimethyl sulfide complex (35 mmol) in dry THF (10 mL), at 70 °C, 24 h, Ar.

**Scheme 3:**
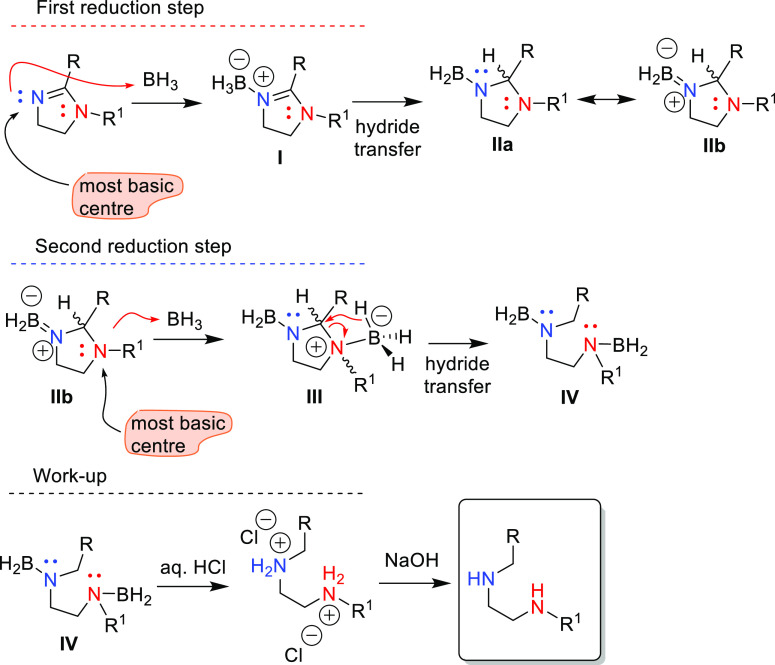
Mechanistic Interpretation of the High Chemoselectivity Found in 2-Imidazoline Reduction with Borane

The excellent chemoselectivity shown by borane dimethyl sulfide, as a reductive agent, can be rationalized by a tentative mechanistic interpretation (Scheme 3) where the reductive opening of 5- or 6-membered cyclic amidines takes place in two consecutive steps, which are directed by the Lewis acid character of borane and its reaction with the most basic centers in reactant or intermediate species.

Thus, as the most basic center in the cyclic amidines is the nitrogen atom at the C(2)=N(3) double bond, the formation of an N(3)-borane adduct, **I**, promotes hydride transfer to C2 to produce the N(3)-imidazolidine-borane intermediate, **II**. For this first hydride transfer, a mechanism similar to that of the well-studied imine reductions is expected, which leads to an intermediate (**II**) that possesses an amine–borane bond.^30^ Such amino-borane structures are defined by a notable double-bond character,^31−33^ as represented by the dipolar canonical form **IIb** in Scheme 3, that also entails a low basicity at N(3) due to the involvement of the nitrogen lone pair in the partial π-bond with boron. Intermediate **II** is crucial to justify the chemoselectivity of the borane reductive opening since the formation of a second amine-boron adduct will now be favored at N(1) position owing to the complete availability of its unshared electron pair which makes of N(1) the most basic center of this intermediate. Consequently, in a second step, the formation of the adduct N(1)-BH_3_ provokes quaternization of N(1) and promotes the hydride transfer to C2 (intermediate **III**), with subsequent imidazolidine opening to give the LSP as the diamine-borane intermediate **IV**. Workup of the crude reaction with aqueous HCl to break N–B bonds, followed by neutralization with NaOH affords the final isolated products.

In addition, in order to explore the feasibility and reliability of this sequential nitrile amidination–reduction strategy for obtaining LSPs, two molecules were selected (spermine and compound **7**) as targets to prove the synthetic approach.

**Scheme 4:**
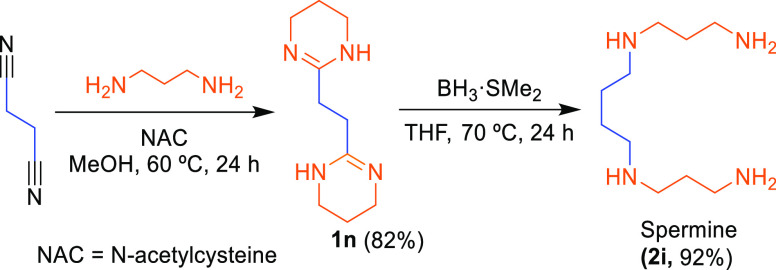
Straightforward Synthesis of Spermine Based on Sequential Nitrile Amidination–Reduction Strategy

First, the synthesis of spermine was carried out through the strategy proposed in Scheme 4. Spermine is a biogenic LSP, currently synthesized by conventional chemical^34,35^ or microbial^36,37^ strategies, which have several practical disadvantages (such as high energy consumption, toxic byproduct generation, heavy environmental pollution, among others). Therefore, it is worth noting that the proposed sequential approach rendered spermine (**2i**) under nonharsh conditions, free of using selective protective groups and tedious workups, in high yield, and without byproduct formation.

**Scheme 5:**
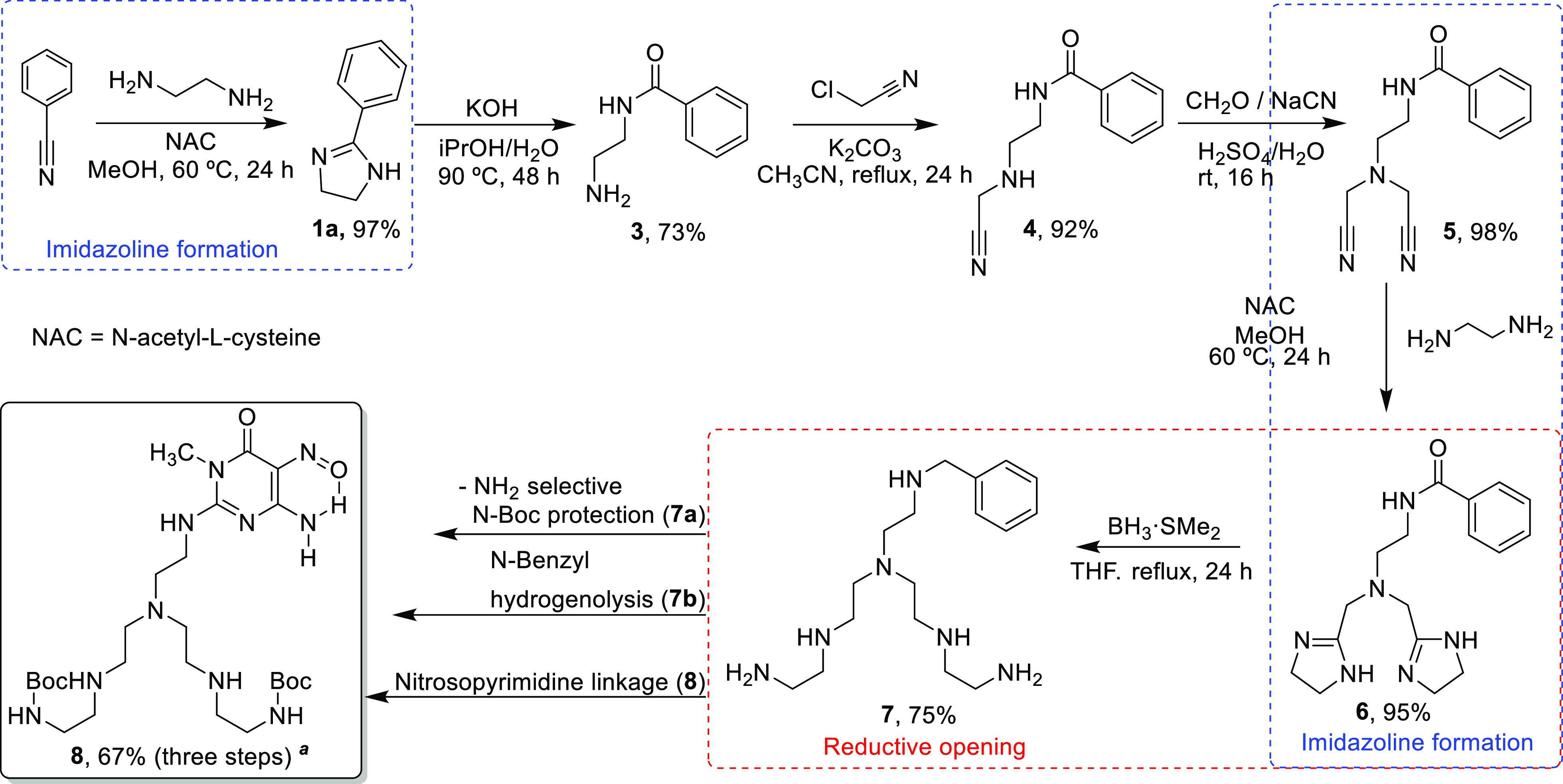
Multistep Synthesis of Branched Polyamine Structures for Pd(II) Receptors

In addition, the potentialities of the nitrile amidination–reduction sequential strategy were proved through the more elaborated synthesis of the branched polyamine structure **7** (Scheme 5), that was prepared during our program to synthesize Pd(II) receptors, like **8**, intended to be incorporated onto a graphene-type surface via noncovalent functionalization for catalytic purposes.^38^

Compound **7** was obtained in high scale, without using additional *N*-protective groups, tedious workups, or purification steps (Scheme 5). Thus, the initial preparation of 2-imidazoline (**1a**) and subsequent hydrolysis provided the *N*-monobenzoylated derivative of ethylenediamine (**3**). Then, a double cyanomethylation on its unprotected nitrogen atom was carried out to afford the corresponding bisnitrile derivate (**5**). Subsequent transformation of its cyano groups into cyclic amidines rendered the double 2-imidazoline (**6**) in good yields and without undesired byproducts. Thus, the reductive opening of both 2-imidazolines with concomitant reduction of the benzamido group afforded the complete skeleton of the branched polyamine structure as the mono-*N*-benzyl derivative (**7**). Finally, three further steps were needed to prepare the Pd(II) receptor **8**.

In conclusion, a straightforward approach for the efficient synthesis of LSPs containing 1,2-diaminoethane and/or 1,3-diaminopropane fragments, based on a nitrile amidination–reduction sequence, was developed under nonharsh conditions, free of using selective protective groups and tedious workups, in high yield, and without byproducts. Thus, the innovative sequence defined by the formation of cyclic amidine derivatives, using *N*-acetylcysteine as an organocatalyst, provided a robust approach to obtain 2-imidazolines, 3,4,5,6-tetrahydropyrimidines, or oxazolines with high yields, under soft conditions, and without undesired byproducts. Besides, their subsequent reduction by borane treatment affords the corresponding linear 1,2-ethylenediamines, 1,3-propylenediamines, or 1,2-hydroxylamine derivatives, respectively, proving that borane is an ideal chemoselective reductive agent for cyclic amidine derivatives.

## Experimental Section

### General Considerations

All reagents were purchased from Aldrich and used without further purification. ^1^H and ^13^C NMR spectra were recorded with a Bruker Advance III (400 MHz) spectrometer with tetramethylsilane as an internal standard. All chemical shifts (δ) are reported in ppm and coupling constants (*J*), in Hz. All chemical shifts are reported relative to tetramethylsilane and d-solvent peaks, respectively. High-resolution mass spectrometry (HRMS) analysis was performed on a Waters Micromass LCT Premier (TOF mass analyzer).

### Synthesis of Single 2-Imidazolines and Derivatives

A round-bottomed flask was charged with the corresponding polyamine (10 mmol, 1.5 equiv) dissolved in MeOH (10 mL, 1.0 mL·mmol^–1^). Then, the nitrile (6.7 mmol, 1 equiv) and *N*-acetylcysteine (10 mmol, 1.5 equiv) were added, and the resulting mixture was heated in an oil bath at 60 °C for 24 h, under an Ar atmosphere. Subsequently, washing with hexane (20 mL) and evaporation of MeOH under vacuum afforded a residual viscous oil that was taken up in an aqueous NaOH solution (15 wt %, 20 mL) and extracted with dichloromethane (2 × 20 mL). Finally, the organic phase was dried over anhydrous Na_2_SO_4_ and concentrated under vacuum to give the final product.

### Synthesis of Double 2-Imidazolines and Derivatives

A round-bottom flask was charged with the corresponding polyamine (10 mmol, 1 equiv) dissolved in MeOH (10 mL, 1.0 mL·mmol^–1^). Then, the nitrile (24 mmol, 2.4 equiv) and *N*-acetylcysteine (10 mmol, 1 equiv) were added and the resulting mixture was heated in an oil bath at 60 °C for 24 h, under an Ar atmosphere. Subsequently, washing with hexane (20 mL) and evaporation of MeOH under vacuum afforded a residual viscous oil that was taken up in an aqueous NaOH solution (15% w/w, 20 mL) and extracted with dichloromethane (2 × 20 mL). Finally, the organic phase was dried over anhydrous Na_2_SO_4_ and concentrated under vacuum to provide the final product.

### Reductive Opening

In a round-bottomed flask, the borane–dimethyl sulfide complex (35 mmol, 1.75 equiv) in dry THF (5 mL, 0.14 mL·mmol^–1^) was introduced. Then, the corresponding imidazoline (10 mmol, 1 equiv) in dry THF (5 mL, 0.5 mL·mmol^–1^) was added into the solution and heated in an oil bath at 70 °C for 24 h, under an Ar atmosphere. Then, the resulting mixture was cooled at room temperature and hydrochloric acid (2 N, 5 mL) was slowly added. The mixture was stirred for 30 min at room temperature. Subsequently, the THF was removed under vacuum and sodium hydroxide pellets were added up to pH 12. Then, the aqueous phase was extracted with dichloromethane (3 × 20 mL). Finally, the organic phase was dried over anhydrous Na_2_SO_4_ and concentrated under vacuum to provide the final product.

#### 2-Phenyl-4,5-dihydro-1*H*-imidazole (**1a**)^39^

Colorless oil, 0.94 g, 97%. ^1^H NMR (400 MHz, CDCl_3_): δ 7.80–7.37 (m, 5H), 4.85 (s, 1H), 3.77 (s, 4H). ^13^C{^1^H} NMR (101 MHz, CDCl_3_): δ 164.8, 130.7, 128.5, 127.0, 50.2. HRMS (ESI/TOF) *m*/*z*: [M + H]^+^ calcd for C_9_H_10_N_2_, 147.0922; found, 147.0916.

#### 2-(2-Phenyl-4,5-dihydro-1*H*-imidazol-1-yl)ethanamine (**1b**)^40^

Yellow oil, 1.18 g, 94%. ^1^H NMR (400 MHz, CDCl_3_): δ 7.67–7.39 (m, 5H), 3.92 (t, *J* = 9.9 Hz, 2H), 3.45 (t, *J* = 9.9 Hz, 2H), 3.10 (t, *J* = 6.4 Hz, 2H), 2.85 (t, *J* = 6.4 Hz, 2H), 1.41 (s, 2H). ^13^C{^1^H} NMR (101 MHz, CDCl_3_): δ 168.1, 132.2, 129.7, 128.4, 128.3, 53.5, 52.6, 51.4, 41.0. HRMS (ESI/TOF) *m*/*z*: [M + H]^+^ calcd for C_11_H_15_N_3_, 190.1344; found, 190.1339.

#### 2-(2-Phenyl-4,5-dihydro-1*H*-imidazol-1-yl)ethanol (**1c**)^41^

Yellow oil, 1.20 g, 95%. ^1^H NMR (400 MHz, CDCl_3_): δ 7.55–7.36 (m, 5H), 3.85 (t, *J* = 9.9 Hz, 2H), 3.61 (t, *J* = 5.8 Hz., 2H), 3.44 (t, *J* = 9.9 Hz, 2H), 3.13 (t, *J* = 5.8 Hz, 2H). ^13^C{^1^H} NMR (CDCl_3_, 101 MHz): δ 168.1, 131.1, 129.8, 128.4, 128.4, 60.4, 53.1, 51.8, 51.5. HRMS (ESI/TOF) *m*/*z*: [M + H]^+^ calcd for C_11_H_14_N_2_O, 191.1184; found, 191.1177.

#### 1,1′-Ethane-1,2-diylbis(2-phenyl-4,5-dihydro-1*H*-imidazole) (**1d**)^42^

Yellow oil, 2.93 g, 92%. ^1^H NMR (400 MHz, CDCl_3_): δ 7.67–7.47 (m, 10H), 3.87 (t, *J* = 9.9 Hz, 4H), 3.29 (t, *J* = 9.9 Hz, 4H), 3.19 (s, 4H). ^13^C{^1^H} NMR (101 MHz, CDCl_3_): δ 167.3, 131.4, 129.8, 128.4, 128.0, 53.5, 51.3, 48.2. HRMS (ESI/TOF) *m*/*z*: [M + H]^+^ calcd for C_20_H_22_N_4_, 319.1923; found, 319.1919.

#### 2-(2-Phenyl-4,5-dihydro-1*H*-imidazol-1-yl)-*N*-[2-(2-phenyl-4,5-dihydro-1*H*-imidazol-1-yl)ethyl]ethanamine (**1e**)

Yellow oil, 3.47 g, 96%. ^1^H NMR (400 MHz, CDCl_3_): δ 7.55–7.35 (m, 10H), 3.88 (t, *J* = 9.9 Hz, 4H), 3.42 (t, *J* = 9.9 Hz, 4H), 3.14 (t, *J* = 6.2 Hz, 4H), 2.72 (t, *J* = 6.2 Hz, 4H), 1.89 (s, 1H). ^13^C{^1^H} NMR (101 MHz, CDCl_3_): δ 167.8, 132.1, 129.7, 128.3, 128.2, 53.4, 51.5, 49.7, 48.6. HRMS (ESI/TOF) *m*/*z*: [M + H]^+^ calcd for C_22_H_27_N_5_, 362.2345; found, 362.2335.

#### 3-(2-Phenyl-4,5-dihydro-1*H*-imidazol-1-yl)propan-1-amine (**1f**)^43^

Yellow oil, 1.24 g, 92%. ^1^H NMR (400 MHz, CDCl_3_): δ 7.51–7.38 (m, 5H), 3.89 (t, *J* = 9.8 Hz, 2H), 3.45 (t, *J* = 9.9 Hz, 2H), 3.09 (t, *J* = 7.0 Hz, 2H), 2.72 (t, *J* = 6.9 Hz, 2H), 1.67 (q, *J* = 7.0 Hz, 2H), 1.26 (s, 2H). ^13^C{^1^H} NMR (101 MHz, CDCl_3_): δ 167.8, 132.1, 129.6, 128.3, 128.0, 53.3, 51.1, 47.1, 39.5, 32.8. HRMS (ESI/TOF) *m*/*z*: [M + H]^+^ calcd for C_12_H_17_N_3_, 204.1501; found, 204.1488.

#### *N*-Benzyl-1-(4,5-dihydro-1*H*-imidazol-2-yl)methanamine (**1g**)

Yellow oil, 1.13 g, 90%. ^1^H NMR (400 MHz, CDCl_3_): δ 7.32–7.28 (m, 5H), 3.79 (s, 2H), 3.57 (s, 4H), 3.43 (s, 2H). ^13^C{^1^H} NMR (101 MHz, CDCl_3_): δ 167.2, 139.8, 128.5, 128.2, 127.2, 53.8, 49.8, 47.3. HRMS (ESI/TOF) *m*/*z*: [M + H]^+^ calcd for C_11_H_15_N_3_, 190.1292; found, 190.1287.

#### 2-{2-[(Benzylamino)methyl]-4,5-dihydro-1*H*-imidazol-1-yl}ethanamine (**1h**)

Yellow oil, 1.41 g, 91%. ^1^H NMR (400 MHz, CDCl_3_): δ 7.34–7.24 (m, 5H), 3.84 (s, 2H), 3.73 (t, *J* = 9.6 Hz, 2H), 3.37 (s, 2H), 3.31 (t, *J* = 9.6 Hz, 2H), 3.10 (t, *J* = 6.1 Hz, 2H), 2.79 (t, *J* = 6.0 Hz, 2H), 1.11 (s, 2H). ^13^C{^1^H} NMR (101 MHz, CDCl_3_): δ 165.9, 139.9, 128.5, 128.4, 127.0, 53.7, 52.5, 50.0, 45.9, 40.6. HRMS (ESI/TOF) *m*/*z*: [M + H]^+^ calcd for C_13_H_20_N_4_, 233.1766; found, 233.1773.

#### 2-Phenyl-3a,4,5,6,7,7a-hexahydro-1*H*-benzimidazole (**1i**)^44^

Yellow oil, 1.22 g, 92%. ^1^H NMR (400 MHz, CDCl_3_): δ 7.78–7.40 (m, 5H), 3.42 (s, 1H), 3.14–3.10 (m, 2H), 2.32–2.29 (m, 2H), 1.86–1.80 (m, 2H), 1.59–1.52 (m, 2H), 1.36 (s, 2H). ^13^C{^1^H} NMR (101 MHz, CDCl_3_): δ 165.4, 130.9, 130.6, 128.4, 126.6, 69.7, 30.9, 25.0. HRMS (ESI/TOF) *m*/*z*: [M + H]^+^ calcd for C_13_H_16_N_2_, 201.1313; found, 201.1329.

#### 2-Phenyl-4,5-dihydro-1,3-oxazole (**1j**)^45^

Yellow oil, 0.93 g, 95%. ^1^H NMR (400 MHz, CDCl_3_): δ 7.94–7.41 (m, 5H), 4.43 (t, *J* = 9.4 Hz, 2H), 4.05 (t, *J* = 9.5 Hz, 2H). ^13^C{^1^H} NMR (101 MHz, CDCl_3_): δ 164.7, 130.4, 128.8, 128.4, 128.2, 67.6, 54.9. HRMS (ESI/TOF) *m*/*z*: [M + H]^+^ calcd for C_9_H_9_NO, 148.0684; found, 148.0699.

#### 3-[2-Phenyl-5,6-dihydropyrimidin-1(4*H*)-yl]propan-1-amine (**1k**)

Yellow oil, 1.34 g, 93%. ^1^H NMR (400 MHz, CDCl_3_): δ 7.55–7.38 (m, 5H), 3.50 (t, *J* = 5.8 Hz, 2H), 3.30 (t, *J* = 6.0 Hz, 2H), 3.07 (t, *J* = 7.2 Hz, 2H), 2.53 (t, *J* = 6.9 Hz, 2H), 1.94 (t, *J* = 5.8 Hz, 2H), 1.59 (q, *J* = 7.0 Hz, 2H), 1.20 (s, 2H). ^13^C{^1^H} NMR (101 MHz, CDCl_3_): δ 159.1, 132.1, 128.4, 128.1, 127.8, 49.7, 45.7, 45.1, 39.3, 32.0, 22.0. HRMS (ESI/TOF) *m*/*z*: [M + H]^+^ calcd for C_13_H_19_N_3_, 218.1657; found, 218.1648.

#### 1,1′-Ethane-1,2-diylbis(2-phenyl-1,4,5,6-tetrahydropyrimidine) (**1l**)

Yellow oil, 3.11 g, 90%. ^1^H NMR (400 MHz, CDCl_3_): δ 7.38–7.25 (m, 10H), 3.45 (t, *J* = 5.6 Hz, 4H), 3.03 (t, *J* = 5.8 Hz, 4H), 2.96 (t, *J* = 5.9 Hz, 4H), 1.83–1.78 (m, 4H). ^13^C{^1^H} NMR (101 MHz, CDCl_3_): δ 158.4, 137.8, 128.5, 128.1, 127.9, 50.1, 46.2, 44.9, 21.8. HRMS (ESI/TOF) *m*/*z*: [M + H]^+^ calcd for C_22_H_26_N_4_, 347.2256; found, 347.2211.

#### 2-Phenyl-1,4,5,6-tetrahydropyrimidine (**1m**)^46^

Yellow oil, 0.87 g, 82%. ^1^H NMR (400 MHz, CDCl_3_): δ 7.66–7.64 (m, 2H), 7.39–7.34 (m, 3H), 3.49 (t, *J* = 5.8 Hz, 4H), 1.85 (quint, *J* = 5.8 Hz, 2H). ^13^C{^1^H} NMR (101 MHz, CDCl_3_): δ 155.1, 137.2, 130.0, 128.6, 126.3, 42.4, 20.9. HRMS (ESI/TOF) *m*/*z*: [M + H]^+^ calcd for C_10_H_12_N_2_, 161.1073; found, 161.1071.

#### 2,2′-Ethane-1,2-diyldi-1,4,5,6-tetrahydropyrimidine (**1n**)

White solid, 0.79 g, 82%. ^1^H NMR (400 MHz, CDCl_3_): δ 3.26 (t, *J* = 5.8 Hz, 8H), 2.36 (s, 4H), 1.71 (quint, *J* = 5.8 Hz, 4H). ^13^C{^1^H} NMR (101 MHz, CDCl_3_): δ 158.6, 41.2, 32.6, 20.4. HRMS (ESI/TOF) *m*/*z*: [M + H]^+^ calcd for C_10_H_18_N_4_, 195.1604; found, 195.1606.

#### *N*-Benzylethane-1,2-diamine (**2a**)^47^

Colorless oil, 1.39 g, 93%. ^1^H NMR (400 MHz, CDCl_3_): δ 7.34–7.26 (m, 5H), 3.80 (s, 2H), 2.80 (t, *J* = 5.8 Hz, 2H), 2.69 (t, *J* = 5.8 Hz, 2H), 1.44 (s, 3H). ^13^C{^1^H} NMR (101 MHz, CDCl_3_): δ 129.4, 128.3, 128.0, 126.8, 53.8, 51.9, 41.7. HRMS (ESI/TOF) *m*/*z*: [M + H]^+^ calcd for C_9_H_14_N_2_, 151.1235; found, 151.1228.

#### *N*-(2-Aminoethyl)-*N*′-benzylethane-1,2-diamine (**2b**)^48^

Colorless oil, 1.87 g, 97%. ^1^H NMR (400 MHz, CDCl_3_): δ 7.30–7.24 (m, 5H), 3.79 (s, 2H), 2.78 (t, *J* = 5.9 Hz, 2H), 2.75–2.74 (m, 4H), 2.65 (t, *J* = 5.8 Hz, 2H), 2.02 (s, 4H). ^13^C{^1^H} NMR (101 MHz, CDCl_3_): δ 140.4, 128.4, 128.2, 127.0, 54.0, 52.4, 49.2, 48.8, 41.7. HRMS (ESI/TOF) *m*/*z*: [M + H]^+^ calcd for C_11_H_19_N_3_, 194.1657; found, 194.1649.

#### *N*-Benzyl-N′-(2-{[2-(benzylamino)ethyl]amino}ethyl)ethane-1,2-diamine (**2c**).^49^

Colorless oil, 2.93 g, 90%. ^1^H NMR (400 MHz, CDCl_3_): δ 7.23–7.31 (m, 10H) 3.78 (s, 4H), 2.74–2.73 (m, 8H), 2.69 (s, 4H), 1.71 (s, 4H). ^13^C{^1^H} NMR (101 MHz, CDCl_3_): δ 140.5, 128.4, 128.1, 126.9, 54.0, 49.4, 48.9. HRMS (ESI/TOF) *m*/*z*: [M + H]^+^ calcd for C_20_H_30_N_4_, 327.2549; found, 327.2545.

#### *N*-Benzyl-N′-{2-[(2-{[2-(benzylamino)ethyl]amino}ethyl) amino]ethyl}ethane-1,2-diamine (**2d**)^50^

Colorless oil, 3.54 g, 96%. ^1^H NMR (400 MHz, CDCl_3_): δ 7.31–7.22 (m, 10H), 3.78 (s, 4H), 2.74–2.72 (m, 8H), 2.71–2.69 (m, 8H), 2.00 (s, 5H). ^13^C{^1^H} NMR (101 MHz, CDCl_3_): δ 140.4, 128.4, 128.1, 126.9, 54.0, 49.4, 49.3, 48.8. HRMS (ESI/TOF) *m*/*z*: [M + H]^+^ calcd for C_22_H_35_N_5_, 370.2955; found, 370.2942.

#### *N*-(2-Aminoethyl)-*N*′-[2-(benzylamino)ethyl]ethane-1,2-diamine (**2e**)^51^

Colorless oil, 2.27 g, 96%. ^1^H NMR (400 MHz, CDCl_3_): δ 7.31–7.24 (m, 5H), 3.78 (s, 2H), 2.82–2.63 (m, 12H), 2.00 (s, 5H). ^13^C{^1^H} NMR (101 MHz, CDCl_3_): δ 140.6, 128.4, 128.1, 126.9, 54.0, 52.6, 49.5, 49.5, 49.4, 49.0, 41.9. HRMS (ESI/TOF) *m*/*z*: [M + H]^+^ calcd for C_13_H_24_N_4_, 237.2079; found, 237.2082.

#### 2-(Benzylamino)ethanol (**2f**)^52^

Colorless oil, 1.40 g, 93%. ^1^H NMR (400 MHz, CDCl_3_): δ 7.31–7.26 (m, 5H), 3.80 (s, 2H), 3.64 (t, *J* = 5.4 Hz, 2H), 2.79 (t, *J* = 5.2 Hz, 2H). ^13^C{^1^H} NMR (101 MHz, CDCl_3_): δ 140.1, 128.5, 128.1, 127.1, 61.0, 53.5, 50.6. HRMS (ESI/TOF) *m*/*z*: [M + H]^+^ calcd for C_9_H_13_NO, 152.0998; found, 152.0991.

#### *N*-(3-Aminopropyl)-*N*′-benzylpropane-1,3-diamine (**2g**)^53^

Colorless oil, 2.19 g, 99%. ^1^H NMR (400 MHz, CDCl_3_): δ 7.24–7.17 (m, 5H), 3.71 (s, 2H), 2.69–2.61 (m, 8H), 1.65–1.57 (m, 4H). ^13^C{^1^H} NMR (101 MHz, CDCl_3_): δ 140.3, 128.3, 128.05, 126.9, 54.0, 48.5, 47.9, 47.8, 40.5, 33.3, 29.9. HRMS (ESI/TOF) *m*/*z*: [M + H]^+^ calcd for C_13_H_23_N_3_, 222.1965; found, 222.1964.

#### *N*-Benzylpropane-1,3-diamine (**2h**)^54^

Colorless oil, 1.56 g, 95%. ^1^H NMR (400 MHz, CDCl_3_): δ 7.23–7.18 (m, 5H), 3.71 (s, 2H), 2.69 (t, *J* = 6.8 Hz, 2H), 2.62 (t, *J* = 7.0 Hz, 2H), 1.58 (quint, *J* = 6.8 Hz, 2H). ^13^C{^1^H} NMR (101 MHz, CDCl_3_): δ 140.3, 128.3, 128.01, 126.8, 54.0, 47.2, 40.5, 33.5. HRMS (ESI/TOF) *m*/*z*: [M + H]^+^ calcd for C_10_H_16_N_2_, 165.1386; found, 165.1384.

#### Spermine (**2i**)^55^

Yellow oil, 0.93 g, 92%. ^1^H NMR (400 MHz, CDCl_3_): δ 2.70 (t, *J* = 7.0 Hz, 4H), 2.60 (t, *J* = 7.0 Hz, 4H), 2.55 (t, *J* = 6.8 Hz, 4H), 1.57 (quint, *J* = 7.0 Hz, 4H), 1.45 (quint, *J* = 6.8 Hz, 4H). ^13^C{^1^H} NMR (101 MHz, CDCl_3_): δ 49.9, 47.8, 40.5, 33.7, 27.8. HRMS (ESI/TOF) *m*/*z*: [M + H]^+^ calcd for C_10_H_26_N_4_, 203.2230; found, 203.2230.

#### *N*-(2-Aminoethyl)benzamide (**3**)^56^

A round-bottom flask was charged with **1a** (7.70 g, 52.67 mmol) dissolved in 2-propanol/H_2_O (1:1 v/v, 160 mL, 3.0 mL·mmol^–1^) with KOH (2.5% weight). The resulting mixture was heated in an oil bath at 95 °C for 48 h. Then, the solvent was removed under vacuum, and the residue was taken up in an aqueous NaOH solution (15% w/w, 50 mL) and extracted with dichloromethane (2 × 30 mL). Finally, the organic phase was dried over anhydrous Na_2_SO_4_ and concentrated under vacuum to give the yellow oil, **3** (6.31 g, 73%). ^1^H NMR (400 MHz, CDCl_3_): δ 7.81–7.78 (m, 2H), 7.49–7.40 (m, 3H), 3.49 (q, *J* = 5.8 Hz, 2H), 2.93 (t, *J* = 6.0 Hz, 2H). ^13^C{^1^H} NMR (101 MHz, CDCl_3_): δ 167.8, 134.7, 131.4, 128.5, 127.0, 42.4, 41.3. HRMS (ESI/TOF) *m*/*z*: [M + H]^+^ calcd for C_9_H_12_N_2_O, 165.1028; found, 165.1023.

#### *N*-{2-[(Cyanomethyl)amino]ethyl}benzamide (**4**)^57^

A round-bottom flask was charged with **3** (1.02 g, 6.24 mmol) dissolved in acetonitrile (20 mL, 3.2 mL·mmol^–1^). Then, K_2_CO_3_ (1.72 g, 12.48 mmol) and chloroacetonitrile (0.6 mL, 9.35 mmol) were added, and the resulting mixture was heated in an oil bath at 60 °C for 24 h, under an Ar atmosphere. Subsequently, the solvent was removed under vacuum, and the residue was taken up in an aqueous NaOH solution (15% w/w, 50 mL) and extracted with dichloromethane (2 × 20 mL). Finally, the organic phase was dried over anhydrous Na_2_SO_4_ and concentrated under vacuum to give the yellow oil, **4** (1.17 g, 92%). ^1^H NMR (400 MHz, CDCl_3_): δ 7.79–7.76 (m, 2H), 7.51–7.40 (m, 3H), 3.62–2.56 (m, 4H), 2.98 (q, *J* = 5.6 Hz, 2H). ^13^C{^1^H} NMR (101 MHz, CDCl_3_): δ 167.9, 134.3, 131.6, 128.6, 126.9, 117.7, 48.1, 39.0, 37.0. HRMS (ESI/TOF) *m*/*z*: [M + H]^+^ calcd for C_11_H_13_N_3_O, 204.1137; found, 204.1130.

#### *N*-{2-[Bis(cyanomethyl)amino]ethyl}benzamide (**5**)

A round-bottomed flask was charged with **4** (2.156 g, 10.61 mmol) dissolved in water (10 mL, 9.4 mL·mmol^–1^) at 0 °C. Then, H_2_SO_4_ (0.62 mL, 11.14 mmol), KCN (0.76 g, 11.14 mmol), and formaldehyde (0.83 mL, 11.14 mmol) were added, and the resulting mixture was stirred for 16 h at room temperature under an Ar atmosphere. Subsequently, an aqueous NaOH solution (15% w/w) was added to the resulting mixture, till a pH of 13 was reached, and extracted with dichloromethane (2 × 20 mL). Finally, the organic phase was dried over anhydrous Na_2_SO_4_ and concentrated under vacuum to give the yellow oil, **5** (2.51 g, 98%). ^1^H NMR (400 MHz, CDCl_3_): δ 7.78–7.76 (m, 2H), 7.54–7.50 (m, 1H), 7.45–7.42 (m, 2H), 3.71 (s, 4H), 3.62 (q, *J* = 6.0 Hz, 2H), 2.92 (t, *J* = 6.0 Hz, 2H). ^13^C{^1^H} NMR (101 MHz, CDCl_3_): δ 168.0, 134.0, 131.8, 128.7, 127.0, 114.3, 53.0, 42.3, 36.7. HRMS (ESI/TOF) *m*/*z*: [M + H]^+^ calcd for C_13_H_14_N_4_O, 243.1246; found, 243.1240.

#### *N*-{2-[Bis(4,5-dihydro-1*H*-imidazol-2-ylmethyl)amino]ethyl}benzamide (**6**)

A round-bottom flask was charged with **5** (3.50 g, 14.45 mmol) dissolved in MeOH (35 mL, 2.4 mL·mmol^–1^). Then, ethylenediamine (7.50 mL, 115.55 mmol) and *N*-acetylcysteine (19.00 g, 115.55 mmol) were added and the resulting mixture was heated in an oil bath at 60 °C for 3 h, under an Ar atmosphere. Subsequently, the *N*-acetylcysteine was removed by extraction with hexane (50 mL) and MeOH was removed under vacuum. The residual oil was taken up in an aqueous NaOH solution (15% w/w, 50 mL) and extracted with dichloromethane (2 × 50 mL). Finally, the organic phase was dried over anhydrous Na_2_SO_4_ and concentrated under vacuum to give the yellow oil, **6** (4.50 g, 95%). ^1^H NMR (400 MHz, CDCl_3_): δ 7.95–7.93 (m, 2H), 7.49–7.45 (m, 1H), 7.43–7.39 (m, 2H), 3.51 (s, 8H), 3.48–3.43 (m, 2H), 3.29 (s, 4H), 2.76 (t, *J* = 5.4 Hz, 2H). ^13^C{^1^H} NMR (101 MHz, CDCl_3_): δ 168.0, 166.9, 134.7, 131.1, 128.2, 137.35, 54.5, 53.2, 49.6, 38.8. HRMS (ESI/TOF) *m*/*z*: [M + H]^+^ calcd for C_17_H_24_N_6_O, 329.2090; found, 329.2081.

#### N′-(2-Aminoethyl)-*N*-{2-[(2-aminoethyl)amino]ethyl}-*N*-[2-(benzylamino)ethyl]ethane-1,2-diamine (**7**)

A round-bottom flask was charged with **6** (3.00 g, 9.13 mmol) dissolved in THF (30 mL, 3.3 mL·mmol^–1^). Then, borane dimethyl sulfide (8.7 mL, 90.13 mmol) in THF (30 mL, 0.3 mL·mmol^–1^) was added into the solution and heated in an oil bath at 70 °C for 24 h, under an Ar atmosphere. Then, the resulting mixture was cooled at room temperature and hydrochloric acid (2 N, 50 mL) was slowly added. The mixture was stirred for 30 min at room temperature. Subsequently, the THF was removed under vacuum and sodium hydroxide pellets were added up to pH 12. Then, the aqueous phase was extracted with dichloromethane (3 × 50 mL). Finally, the organic phase was dried over anhydrous Na_2_SO_4_ and concentrated under vacuum to give the colorless oil, **7** (2.19 g, 75%). ^1^H NMR (400 MHz, CDCl_3_): δ 7.32–7.26 (m, 5H), 3.78 (s, 2H), 2.76–2.57 (m, 20H), 2.50 (s, 7H). ^13^C{^1^H} NMR (101 MHz, CDCl_3_): δ 140.2, 128.4, 128.2, 127.05, 54.2, 54.1, 54.0, 47.3, 47.0, 41.4. HRMS (ESI/TOF) *m*/*z*: [M + H]^+^ calcd for C_17_H_34_N_6_, 323.2917; found, 323.2916.

#### Di-*tert*-butyl{[({[2-(benzylamino)ethyl]azanediyl}bis(ethane-2,1-diyl))bis(azanediyl)]bis(ethane-2,1-diyl)}dicarbamate (**7a**)

A round-bottom flask was charged with **7** (5.00 g, 15.50 mmol) dissolved in toluene (100 mL, 6.4 mL·mmol^–1^). Then, 1-Boc-imidazole (5.50 g, 32.56 mmol) was added into the solution and heated in an oil bath at 60 °C for 5 h, under an Ar atmosphere. Then, the solvent is removed under vacuum and the resulting mixture was taken up in an aqueous NaOH solution (15% w/w, 50 mL) and extracted with dichloromethane (2 × 50 mL). The organic phase is concentrated under vacuum to give the yellow oil **7a** (6.64 g, 82%). ^1^H NMR (400 MHz, CDCl_3_): δ 7.33–7.30 (m, 4H), 7.27–7.23 (m, 1H), 3.81 (s, 2H), 3.20–3.16 (m, 4H), 2.69 (t, *J* = 5.8 Hz, 4H), 2.68 (t, *J* = 5.8 Hz, 2H), 2.64 (t, *J* = 6.2 Hz, 4H), 2.57 (t, *J* = 5.8 Hz, 2H), 2.53 (t, *J* = 5.8 Hz, 4H), 1.44 (s, 18H). ^13^C{^1^H} NMR (101 MHz, CDCl_3_): δ 156.15, 140.15, 128.4, 128.1, 127.0, 79.0, 53.9, 53.7, 53.7, 49.0, 46.8, 46.6, 40.0, 28.4. HRMS (ESI/TOF) *m*/*z*: [M + H]^+^ calcd for C_27_H_50_N_6_O_4_, 523.3972; found, 523.3968.

#### Di-*tert*-butyl[({[(2-aminoethyl)azanediyl]bis(ethane-2,1-diyl)}bis(azanediyl))bis(ethane-2,1-diyl)]dicarbamate (**7b**)

A round-bottom flask was charged with **7a** (2.90 g, 5.54 mmol) dissolved in EtOH (100 mL, 18.0 mL·mmol^–1^). Then, Pd/C (8.00 g, 10% Pd) was added into the solution and kept stirring for 24 h at room temperature under a H_2_ atmosphere. Then, the resulting mixture is filtered in order to remove the catalyst, and the organic phase is concentrated under vacuum to give the yellow oil, **7b** (1.98 g, 83%). ^1^H NMR (400 MHz, CDCl_3_): δ 3.27–3.21 (m, 4H), 2.77 (t, *J* = 6.0 Hz, 2H), 2.74 (t, *J* = 5.4 Hz, 4H), 2.68 (t, *J* = 5.8 Hz, 4H), 2.56 (t, *J* = 6.0 Hz, 4H), 2.49 (t, *J* = 6.0 Hz, 2H), 1.44 (s, 18H). ^13^C{^1^H} NMR (101 MHz, CDCl_3_): δ 156.3, 79.05, 56.4, 53.6, 49.1, 46.7, 40.0, 39.4, 28.5. HRMS (ESI/TOF) *m*/*z*: [M + H]^+^ calcd for C_20_H_44_N_6_O_4_, 433.3502; found, 433.3498.

#### Di-*tert*-butyl({[({2-[(4-amino-1-methyl-5-nitroso-6-oxo-1,6-dihydropyrimidin-2-yl)amino]ethyl}azanediyl)bis(ethane-2,1-diyl)]bis(azanediyl)}bis(ethane-2,1-diyl))dicarbamate (**8**)

A round-bottomed flask was charged with **7b** (1.49 g, 3.45 mmol) dissolved in MeOH (20 mL, 5.8 mL·mmol^–1^). Then, 6-amino-1-methyl-2-metoxi-5-nitrosopyrimidin-4(3*H*)-one (0.80 g, 3.96 mmol) was added to the solution, and the mixture was heated in an oil bath at 45 °C for 6 h. Then, the resulting mixture is filtered in order to remove the solid residue (excess 2,6-diamino-1-methyl-5-nitrosopyrimidin-4(3*H*)-one) and the organic phase is concentrated under vacuum to give the red solid, **8** (2.00 g, 99%). ^1^H NMR (400 MHz, DMSO-*d*_6_): δ 3.44 (t, *J* = 6.6 Hz, 2H), 3.33 (s, 3H), 3.00–2.95 (m, 4H), 2.62 (t, *J* = 6.6 Hz, 2H), 2.56–2.50 (m, 12H), 1.35 (s, 18H). ^13^C{^1^H} NMR (101 MHz, DMSO-*d*_6_): δ 161.5, 155.6, 154.4, 149.9, 142.0, 77.5, 53.6, 52.6, 48.9, 46.9, 39.6, 39.0, 28.2, 27.2. HRMS (ESI/TOF) *m*/*z*: [M + H]^+^ calcd for C_25_H_48_N_10_O_6_, 585.3837; found, 585.3834.

## Data Availability

The data underlying this study are available in the published article and its Supporting Information.

## Supporting Information Available

Multistep synthesis of compound **8** and copies of NMR spectra of compounds (PDF)

## References

[ref1] Minois N. Molecular Basis of the ‘Anti-Aging’’ Effect of Spermidine and Other Natural Polyamines - A Mini-Review’. Gerontology 2014, 60, 319–326. 10.1159/000356748. 24481223

[ref2] Moinard C.; Cynober L.; de Bandt J.-P. Polyamines: metabolism and implications in human diseases. Clin. Nutr. 2005, 24, 184–197. 10.1016/j.clnu.2004.11.001. 15784477

[ref3] Karigiannis G.; Papaioannou D. Structure, Biological Activity and Synthesis of Polyamine Analogues and Conjugates. Eur. J. Org Chem. 2000, 2000, 1841–1863. 10.1002/(SICI)1099-0690(200005)2000:10<1841::AID-EJOC1841>3.0.CO;2-9.

[ref4] Igarashi K.; Kashiwagi K. Modulation of cellular function by polyamines. Int. J. Biochem. Cell Biol. 2010, 42, 39–51. 10.1016/j.biocel.2009.07.009. 19643201

[ref5] Huang Y.; Pledgie A.; Casero R. A. J.; Davidson N. E. Molecular mechanisms of polyamine analogs in cancer cells. Anticancer Drugs 2005, 16, 229–241. 10.1097/00001813-200503000-00002. 15711175

[ref6] Wilson D.; Boyle G. M.; McIntyre L.; Nolan M. J.; Parsons P. G.; Smith J. J.; Tribolet L.; Loukas A.; Liddell M. J.; Rash L. D.; Daly N. L. The Aromatic Head Group of Spider Toxin Polyamines Influences Toxicity to Cancer Cells. Toxins 2017, 9, 346 10.3390/toxins9110346. 29077051 PMC5705961

[ref7] Hofer S. J.; Simon A. K.; Bergmann M.; Eisenberg T.; Kroemer G.; Madeo F. Mechanisms of spermidine-induced autophagy and geroprotection. Nat. Aging 2022, 2, 1112–1129. 10.1038/s43587-022-00322-9. 37118547

[ref8] Gilmore J. L.; Yi X.; Quan L.; Kabanov A. V. Novel Nanomaterials for Clinical Neuroscience. J. Neuroimmune Pharmacol. 2008, 3, 83–94. 10.1007/s11481-007-9099-6. 18210200 PMC2566785

[ref9] Mäkitie L. T.; Kanerva K.; Polvikoski T.; Paetau A.; Andersson L. C. Brain Neurons Express Ornithine Decarboxylase-Activating Antizyme Inhibitor 2 with Accumulation in Alzheimer’s Disease. Brain Pathol. 2010, 20, 571–580. 10.1111/j.1750-3639.2009.00334.x. 19832840 PMC8094758

[ref10] Alkhzem A. H.; Li S.; Wonfor T.; Woodman T. J.; Laabei M.; Blagbrough I. S. Practical Synthesis of Antimicrobial Long Linear Polyamine Succinamides. ACS Bio Med Chem Au 2022, 2, 607–616. 10.1021/acsbiomedchemau.2c00033. PMC10125363 37101429

[ref11] Uchida H.; Itaka K.; Uchida S.; Ishii T.; Suma T.; Miyata K.; Oba M.; Nishiyama N.; Kataoka K. Synthetic Polyamines to Regulate mRNA Translation through the Preservative Binding of Eukaryotic Initiation Factor 4E to the Cap Structure. J. Am. Chem. Soc. 2016, 138, 1478–1481. 10.1021/jacs.5b11726. 26811205

[ref12] Li B.; Zhang X.; Dong Y. Nanoscale platforms for messenger RNA delivery. Wiley Interdiscip. Rev.: Nanomed. Nanobiotechnol. 2019, 11, e1530 10.1002/wnan.1530. 29726120 PMC6443240

[ref13] Jarzębińska A.; Pasewald T.; Lambrecht J.; Mykhaylyk O.; Kümmerling L.; Beck P.; Hasenpusch G.; Rudolph C.; Plank C.; Dohmen C. A Single Methylene Group in Oligoalkylamine-Based Cationic Polymers and Lipids Promotes Enhanced mRNA Delivery. Angew. Chem., Int. Ed. 2016, 55, 9591–9595. 10.1002/anie.201603648. 27376704

[ref14] Akinc A.; Zumbuehl A.; Goldberg M.; Leshchiner E. S.; Busini V.; Hossain N.; Bacallado S. A.; Nguyen D. N.; Fuller J.; Alvarez R.; Borodovsky A.; Borland T.; Constien R.; de Fougerolles A.; Dorkin J. R.; Narayanannair Jayaprakash K.; Jayaraman M.; John M.; Koteliansky V.; Manoharan M.; Nechev L.; Qin J.; Racie T.; Raitcheva D.; Rajeev K. G.; Sah D. W. Y.; Soutschek J.; Toudjarska I.; Vornlocher H.-P.; Zimmermann T. S.; Langer R.; Anderson D. G. A combinatorial library of lipid-like materials for delivery of RNAi therapeutics. Nat. Biotechnol. 2008, 26, 561–569. 10.1038/nbt1402. 18438401 PMC3014085

[ref15] Firpo M. R.; Mastrodomenico V.; Hawkins G. M.; Prot M.; Levillayer L.; Gallagher T.; Simon-Loriere E.; Mounce B. C. Targeting Polyamines Inhibits Coronavirus Infection by Reducing Cellular Attachment and Entry. ACS Infect. Dis. 2021, 7, 1423–1432. 10.1021/acsinfecdis.0c00491. 32966040 PMC7539557

[ref16] Bargeman G. Recent developments in the preparation of improved nanofiltration membranes for extreme pH conditions. Sep. Purif. Technol. 2021, 279, 119725 10.1016/j.seppur.2021.119725.

[ref17] Zhang H.; Zhang X.; Bao C.; Li X.; Duan F.; Friedrich K.; Yang J. Skin-Inspired, Fully Autonomous Self-Warning and Self-Repairing Polymeric Material under Damaging Events. Chem. Mater. 2019, 31, 2611–2618. 10.1021/acs.chemmater.9b00398.

[ref18] Zhang H.; Zhang X.; Bao C.; Li X.; Sun D.; Duan F.; Friedrich K.; Yang J. Direct microencapsulation of pure polyamine by integrating microfluidic emulsion and interfacial polymerization for practical self-healing materials. J. Mater. Chem. A 2018, 6, 24092–24099. 10.1039/C8TA08324J.

[ref19] Verdejo B.; Inclán M.; Clares M. P.; Bonastre-Sabater I.; Ruiz-Gasent M.; García-España E. Fluorescent Chemosensors Based on Polyamine Ligands: A Review. Chemosensors 2021, 10, 1 10.3390/chemosensors10010001.

[ref20] Kuksa V.; Buchan R.; Kong Thoo Lin P. Synthesis of Polyamines, Their Derivatives, Analogues and Conjugates. Synthesis 2000, 2000, 1189–1207. 10.1055/s-2000-6405.

[ref21] Lange U. E. W.; Schäfer B.; Baucke D.; Buschmann E.; Mack H. A new mild method for the synthesis of amidines. Tetrahedron Lett. 1999, 40, 7067–7070. 10.1016/S0040-4039(99)01460-4.

[ref22] Yamamoto H.; Maruoka K. Regioselective carbonyl amination using diisobutylaluminum hydride. J. Am. Chem. Soc. 1981, 103, 4186–4194. 10.1021/ja00404a035.

[ref23] Zhen S.; Zheng L.; Yinjuan B. The reduction ring-opening reaction of imidazoline. Sci. China, Ser. B: Chem. 2000, 43, 69–75. 10.1007/BF03028851.

[ref24] Hofmann A. W. Notiz uber Anhydrobasen der aliphatischen Diamine. Chem. Ges. 1888, 21, 2332–2338. 10.1002/cber.18880210228.

[ref25] Mehedi M. S. A.; Tepe J. J. Recent Advances in the Synthesis of Imidazolines (2009–2020). Adv. Synth. Catal. 2020, 362, 4189–4225. 10.1002/adsc.202000709.

[ref26] Liu H.; Du D.-M. Recent Advances in the Synthesis of 2-Imidazolines and Their Applications in Homogeneous Catalysis. Adv. Synth. Catal. 2009, 351, 489–519. 10.1002/adsc.200800797.

[ref27] Sapegin A.; Krasavin M. Ring-opening reactions of 2-imidazolines and their applications. Adv. Heterocycl. Chem. 2020, 130, 195–250. 10.1016/bs.aihch.2019.10.004.

[ref28] Schnur R. C. A generalized and proton-catalyzed synthesis of amidines from thioimidates. J. Org. Chem. 1979, 44, 3726–3727. 10.1021/jo01335a028.

[ref29] Zaidlewicz M.; Baum O.; Srebnik M. Borane Dimethyl Sulfide. Encyclopedia of Reagents for Organic Synthesis; Wiley Online Library, 2006.

[ref30] Zhai S.; Vidović D.; Petković M. Hydroboration of imines: intermolecular vs. intramolecular hydride transfer. New J. Chem. 2023, 47, 11544–11556. 10.1039/D3NJ01979A.

[ref31] Staubitz A.; Besora M.; Harvey J. N.; Manners I. Computational Analysis of Amine-Borane Adducts as Potential Hydrogen Storage Materials with Reversible Hydrogen Uptake. Inorg. Chem. 2008, 47, 5910–5918. 10.1021/ic800344h. 18500797

[ref32] Robertson A. P. M.; Leitao E. M.; Manners I. Catalytic Redistribution and Polymerization of Diborazanes: Unexpected Observation of Metal-Free Hydrogen Transfer between Aminoboranes and Amine-Boranes. J. Am. Chem. Soc. 2011, 133, 19322–19325. 10.1021/ja208752w. 22035112

[ref33] Zou W.; Gao L.; Cao J.; Li Z.; Li G.; Wang G.; Li S. Mechanistic Insight into Hydroboration of Imines from Combined Computational and Experimental Studies. Chem. - Eur. J. 2022, 28, e202104004 10.1002/chem.202104004. 35018677

[ref34] Almeida M. L. S.; Grehn L.; Ragnarsson U. Selective protection of polyamines: synthesis of model compounds and spermidine derivatives. J. Chem. Soc., Perkin Trans. 1 1988, 1905–1911. 10.1039/p19880001905.

[ref35] Covassin L.; Desjardins M.; Charest-Gaudreault R.; Audette M.; Bonneau M.-J.; Poulin R. Synthesis of spermidine and norspermidine dimers as high affinity polyamine transport inhibitors. Bioorg. Med. Chem. Lett. 1999, 9, 1709–1714. 10.1016/S0960-894X(99)00262-0. 10397506

[ref36] Zou D.; Li L.; Min Y.; Ji A.; Liu Y.; Wei X.; Wang J.; Wen Z. Biosynthesis of a Novel Bioactive Metabolite of Spermidine from Bacillus amyloliquefaciens: Gene Mining, Sequence Analysis, and Combined Expression. J. Agric. Food Chem. 2021, 69, 267–274. 10.1021/acs.jafc.0c07143. 33356220

[ref37] Zou D.; Zhao Z.; Li L.; Min Y.; Zhang D.; Ji A.; Jiang C.; Wei X.; Wu X. A comprehensive review of spermidine: Safety, health effects, absorption and metabolism, food materials evaluation, physical and chemical processing, and bioprocessing. Compr. Rev. Food Sci. Food Saf. 2022, 21, 2820–2842. 10.1111/1541-4337.12963. 35478379

[ref38] Valbuena-Rus A. M.; Gutiérrez-Valero M. D.; Arranz-Mascarós P.; López-Garzón R.; Melguizo M.; Vernet-García J.; Pérez-Mendoza M.; Godino-Salido M. L. Synergy of semiconductor components of non-covalent functionalized (PdS doped)-G CdS NPs composite provide efficient photocatalytic water reduction under visible light. Appl. Surf. Sci. 2021, 554, 149646 10.1016/j.apsusc.2021.149646.

[ref39] Manikandan R.; Anitha P.; Prakash G.; Vijayan P.; Viswanathamurthi P.; Butcher R. J.; Malecki J. G. Ruthenium(II) carbonyl complexes containing pyridoxal thiosemicarbazone and trans-bis(triphenylphosphine/arsine): Synthesis, structure and their recyclable catalysis of nitriles to amides and synthesis of imidazolines. J. Mol. Catal. A: Chem. 2015, 398, 312–324. 10.1016/j.molcata.2014.12.017.

[ref40] Chen B.; Chen Y.; Huang Z.; Xiong G.; Yao X.; Cao M. Preparation method of sodium p-nitrophenolate in the presence of quaternary ammonium surfactant. CN 113200862 A, 2021.

[ref41] Zhang J.; Wang X.; Yang M.; Wan K.; Yin B.; Wang Y.; Li J.; Shi Z. Copper-catalyzed synthesis of 2-imidazolines and their N-hydroxyethyl derivatives under various conditions. Tetrahedron Lett. 2011, 52, 1578–1582. 10.1016/j.tetlet.2011.01.082.

[ref42] Marxer A. The Acylation and Alkylation of Imidazolines and Some New Types of Imidazolines^1^. J. Am. Chem. Soc. 1957, 79, 467–472. 10.1021/ja01559a065.

[ref43] Sawa N.; Okamura S. Synthesis of N-(Cyanoethyl)imidazolines and Distinction between their 1, 4- and 1, 5-Isomers. Nippon kagaku zassi 1970, 91, 288–291. 10.1246/nikkashi1948.91.3_288.

[ref44] Loughlin W. A.; Jenkins I. D.; Petersson M. J. Cyclodehydration of N-(Aminoalkyl)benzamides under Mild Conditions with a Hendrickson Reagent Analogue. J. Org. Chem. 2013, 78, 7356–7361. 10.1021/jo401082q. 23805907

[ref45] Trose M.; Lazreg F.; Lesieur M.; Cazin C. S. J. Copper N-Heterocyclic Carbene Complexes As Active Catalysts for the Synthesis of 2-Substituted Oxazolines from Nitriles and Aminoalcohols. J. Org. Chem. 2015, 80, 9910–9914. 10.1021/acs.joc.5b01382. 26423118

[ref46] Tian X.; Song L.; Li E.; Wang Q.; Yu W.; Chang J. Metal-free one-pot synthesis of 1,3-diazaheterocyclic compounds via I2-mediated oxidative C-N bond formation. RSC Adv. 2015, 5, 62194–62201. 10.1039/C5RA11262A.

[ref47] Micklitsch C. M.; Yu Q.; Schneider J. P. Unnatural multidentate metal ligating α-amino acids. Tetrahedron Lett. 2006, 47, 6277–6280. 10.1016/j.tetlet.2006.06.128.

[ref48] Ernst M.; Altenhoff A. G.; Luyken H.; Huber T.; Haupt S.; Kolassa D. Process for preparing amines over a copper catalyst. WO 2021110472 A1, 2021.

[ref49] Bechara G.; Leygue N.; Galaup C.; Mestre B.; Picard C. An efficient route to pyridine and 2,2’-bipyridine macrocycles incorporating a triethylenetetraminetetraacetic acid core as ligand for lanthanide ions. Tetrahedron Lett. 2009, 50, 6522–6525. 10.1016/j.tetlet.2009.09.030.

[ref50] Bernardo M. A.; Guerrero J. A.; García-España E.; Luis S. V.; Llinares J. M.; Pina F.; Ramírez J. A.; Soriano C. Thermodynamic, NMR and photochemical study on the acid–base behaviour of N,N′-dibenzylated polyamines and on their interaction with hexacyanocobaltate(III). J. Chem. Soc., Perkin Trans. 2 1996, 2335–2342. 10.1039/P29960002335.

[ref51] Burdinski D.; Lub J.; Pikkemaat J. A.; Moreno Jalón D.; Martial S.; Del Pozo Ochoa C. Triethylenetetramine penta- and hexa-acetamide ligands and their ytterbium complexes as paraCEST contrast agents for MRI. Dalton Trans. 2008, 4138–4151. 10.1039/b803557a. 18688432

[ref52] Zhang Z.; Ikeda T.; Murayama H.; Honma T.; Tokunaga M.; Motoyama Y. Anchored Palladium Complex-Generated Clusters on Zirconia: Efficiency in Reductive N-Alkylation of Amines with Carbonyl Compounds under Hydrogen Atmosphere. Chem. - Asian J. 2022, 17, e202101243 10.1002/asia.202101243. 35266303

[ref53] Petitjean A.; Cuccia L. A.; Schmutz M.; Lehn J.-M. Naphthyridine-Based Helical Foldamers and Macrocycles: Synthesis, Cation Binding, and Supramolecular Assemblies. J. Org. Chem. 2008, 73, 2481–2495. 10.1021/jo702495u. 18315005

[ref54] Tong-Tao X.; Jian G.; Xing-You X.; Xu-Jie Y.; Lu-De L.; Wang X. Synthesis, structure and antimicrobial study of two copper(II) complexes derived from paeonol and R-NH-propyldiamine. J. Coord. Chem. 2007, 60, 1721–1729. 10.1080/00958970601117365.

[ref55] Chen J.; Ni H.; Meng Z.; Wang J.; Huang X.; Dong Y.; Sun C.; Zhang Y.; Cui L.; Li J.; Jia X.; Meng Q.; Li C. Supramolecular trap for catching polyamines in cells as an anti-tumor strategy. Nat. Commun. 2019, 10, 3546–3548. 10.1038/s41467-019-11553-7. 31391464 PMC6685945

[ref56] Rebelo R. A.; Rezende M. C.; Nome F.; Zucco C. The Use of 2,2,2-Trichloro-1-Arylethanones as Benzoylating Agents. Synth. Commun. 1987, 17, 1741–1748. 10.1080/00397918708063993.

[ref57] Das S. K.; Venkaiahgari V.; Ravinder B.; Dubey M. K.; Bandichhor R. Novel route of synthesis of some trientine impurities and their characterization. DerPharmaChemica 2020, 13, 23–31.

