# Supplementary material for: Sequential Nitrile Amidination–Reduction as a Straightforward Procedure to Selective Linear Polyamine Preparation
Source: J Org Chem. 2023 Nov 25;88(24):17274–83. doi: 10.1021/acs.joc.3c02128 (PMC10729039; doi:10.1021/acs.joc.3c02128)

Supporting information

Sequential nitrile amidination–reduction as a straightforward procedure to selective primary benzylation or linear polyamine extension

Antonio Peñas-Sanjuán, Jose J. Chica-Armenteros, Rubén Cruz-Sánchez, Celeste García-Gallarín and Manuel Melguizo*

Table of Contents

1. Multi-step synthesis of compound **8**…………………………………………………………………S1

2. NMR Spectra of the Compounds……………………..……………………………………………………..S2

1. Multi-step synthesis of compound 8

Synthesis of di-tert-butyl (((((2-((4-amino-1-methyl-5-nitroso-6-oxo-1,6-dihydropyrimidin-2-yl)amino)ethyl)azanediyl)bis(ethane-2,1-diyl))bis(azanediyl))bis(ethane-2,1-diyl))dicarbamate (8)

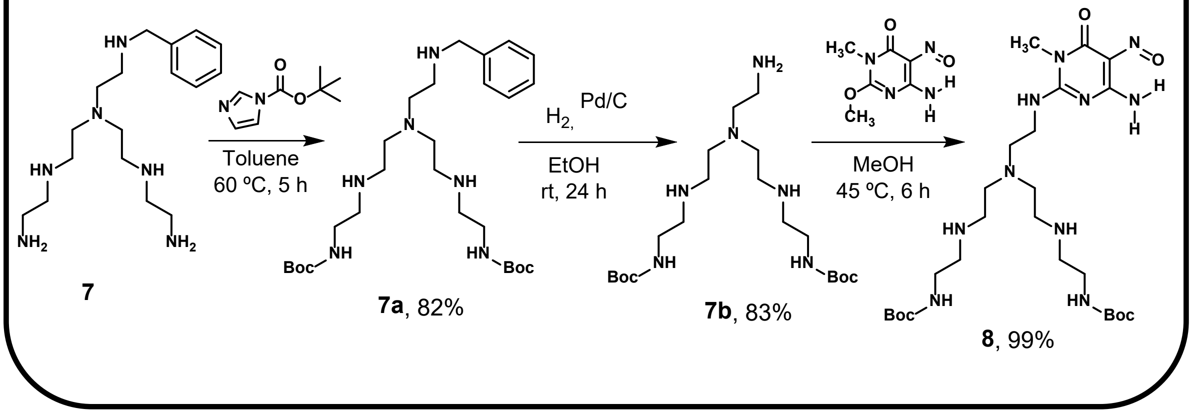

2. NMR Spectra of the Compounds

Fig. S1. The ^1H (400MHz) and ^{13}C (101MHz) NMR spectra for 2-phenyl-4,5-dihydro-1H-imidazole (1a) in $CDCl_3$

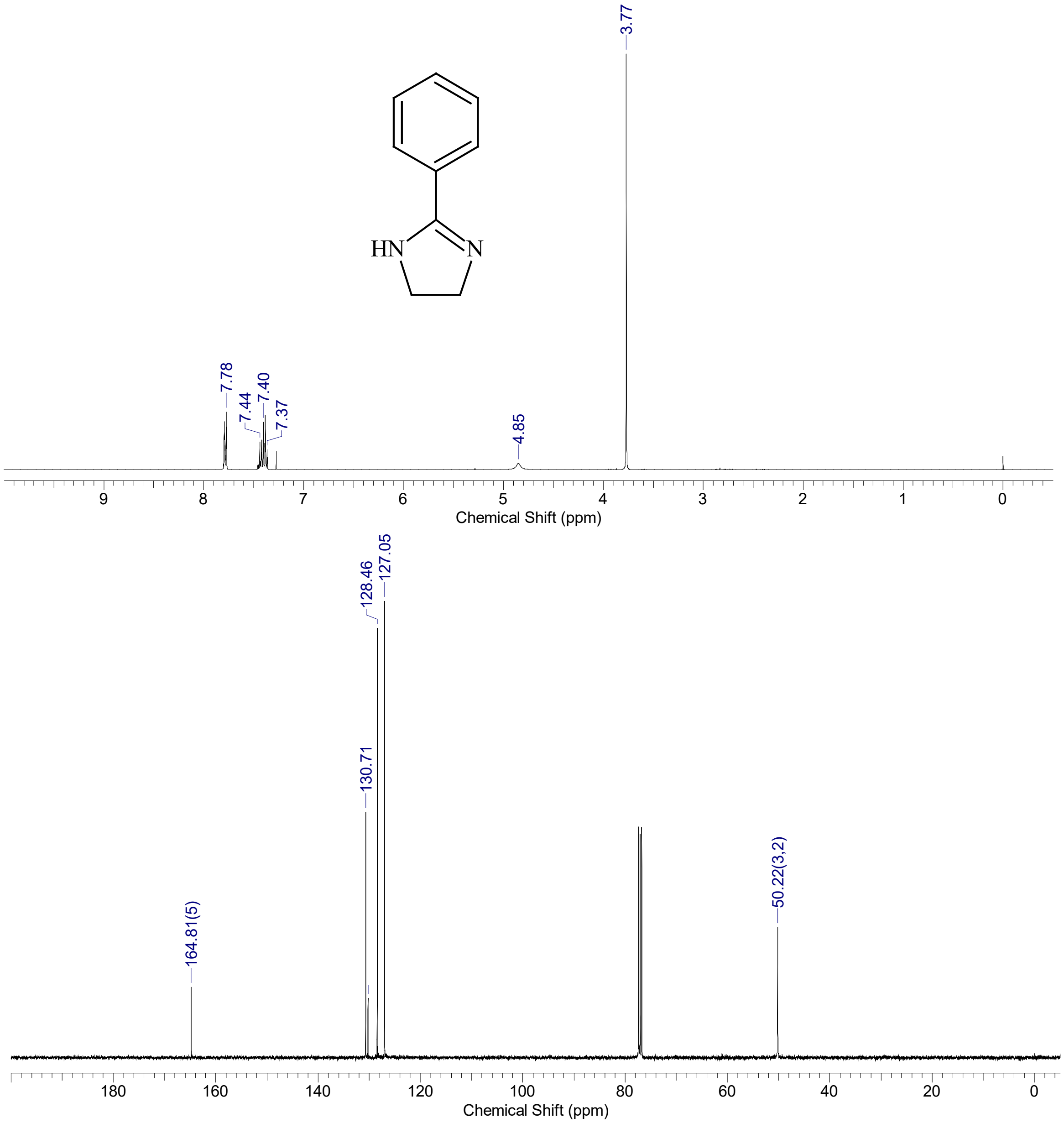

Fig. S2. The ¹H (400MHz) and ¹³C (101MHz) NMR spectra for 2-(2-phenyl-4,5-dihydro-1H-imidazol-1-yl)ethanamine (1b) in $CDCl_3$

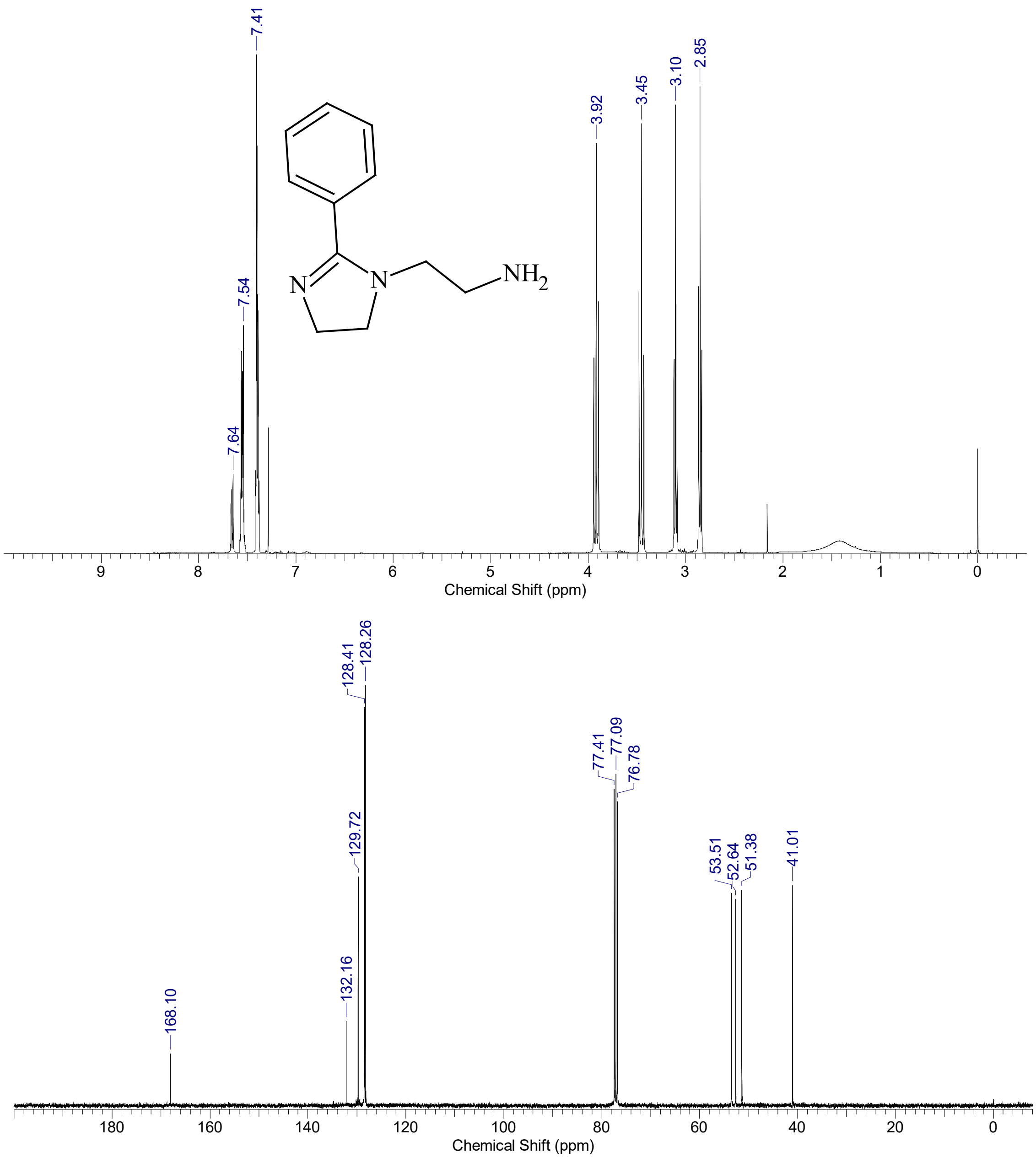

Fig. S3. The ^{1}H (400MHz) and ^{13}C (101MHz) NMR spectra for 2-(2-phenyl-4,5-dihydro-1H-imidazol-1-yl)ethanol (1c) in $CDCl_3$

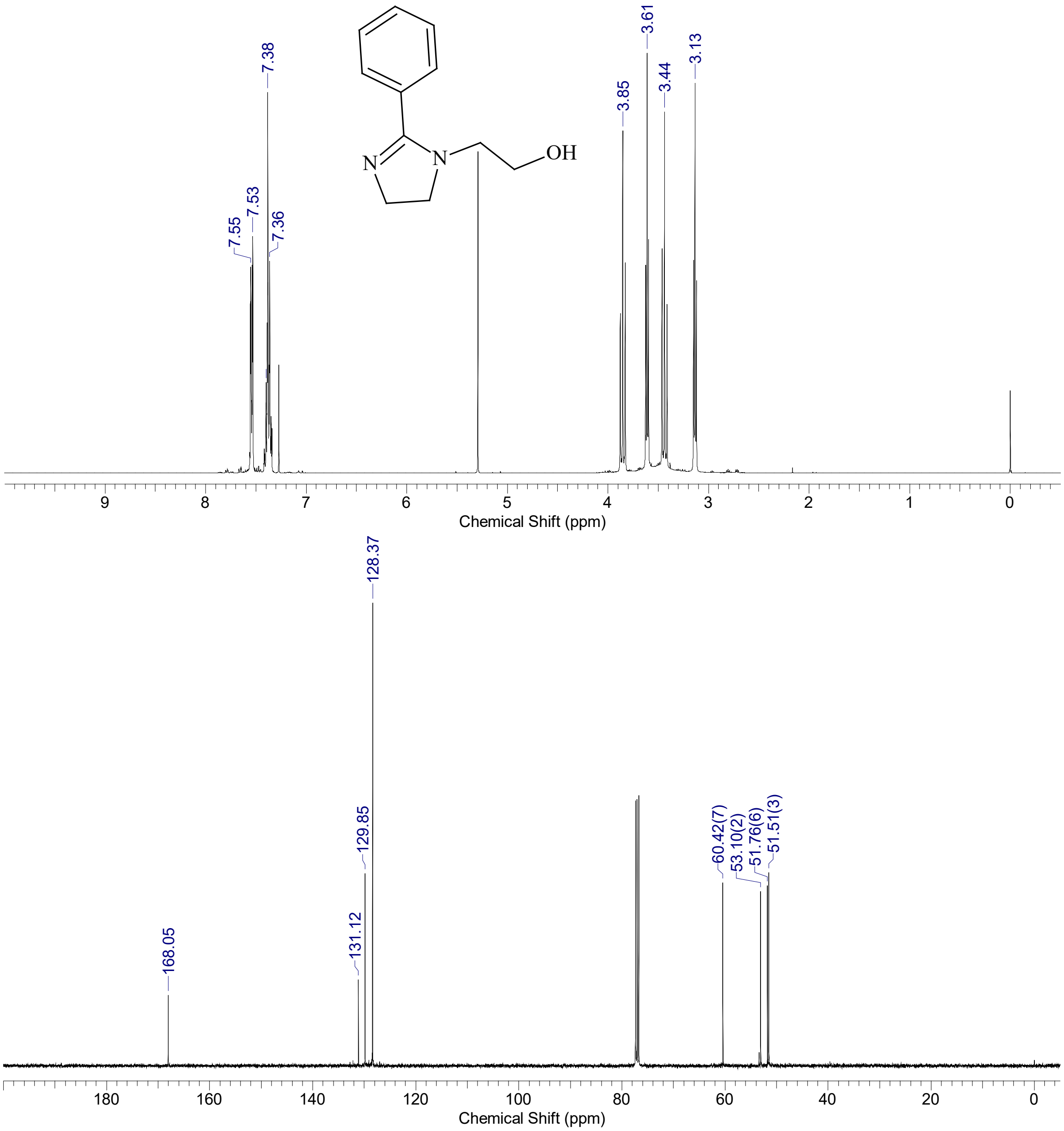

Fig. S4. The ^{1}H (400MHz) and ^{13}C (101MHz) NMR spectra for 1,1'-ethane-1,2-diylbis(2-phenyl-4,5-dihydro-1H-imidazole) (1d) in $CDCl_3$

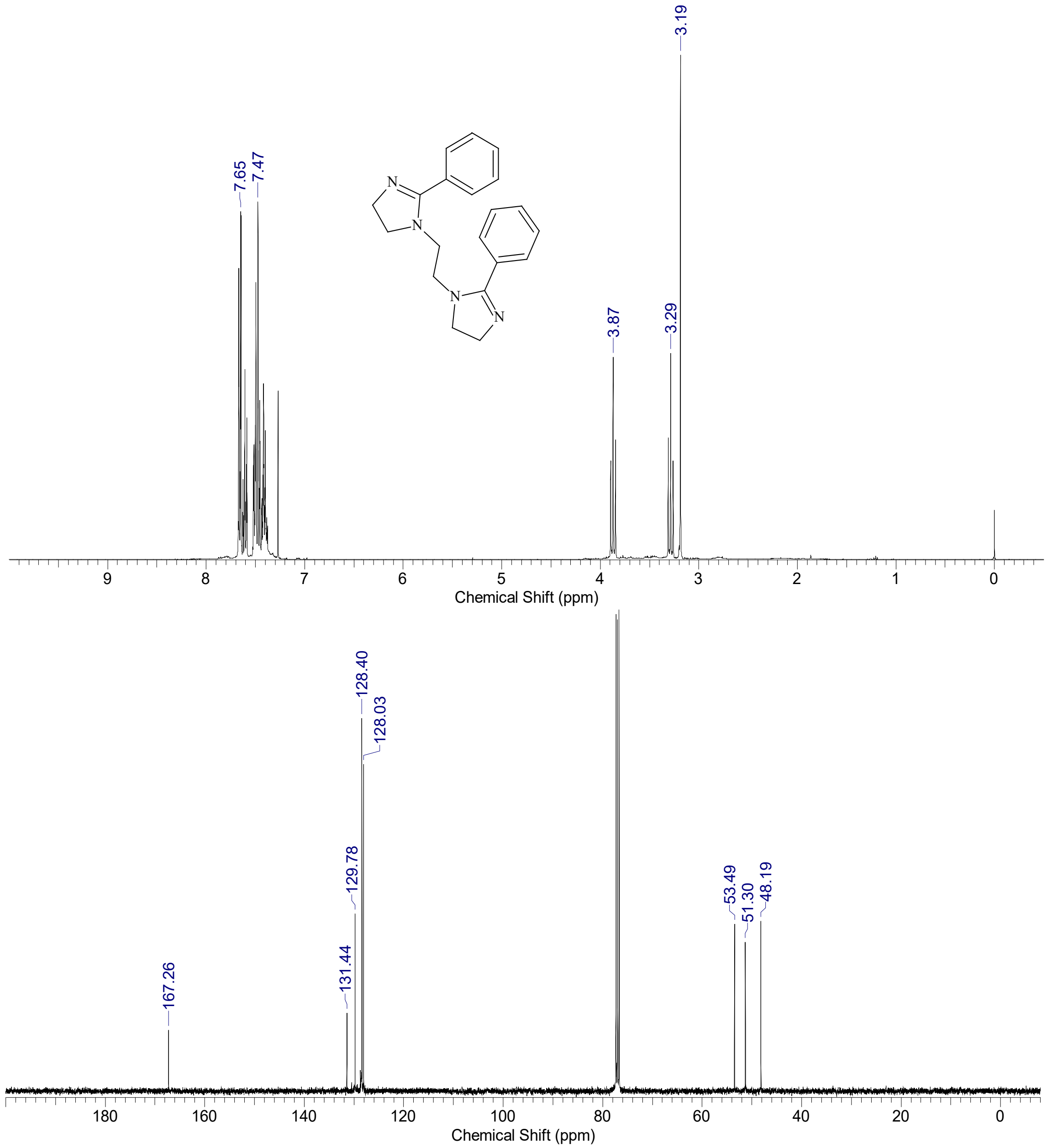

Fig. S5. The ^{1}H (400MHz) and ^{13}C (101MHz) NMR spectra for 2-(2-phenyl-4, 5-dihydro-1H-imidazol-1-yl)-N-[2-(2-phenyl-4, 5-dihydro-1H-imidazol-1-yl)ethyl]ethanamine (1e) in $CDCl_3$

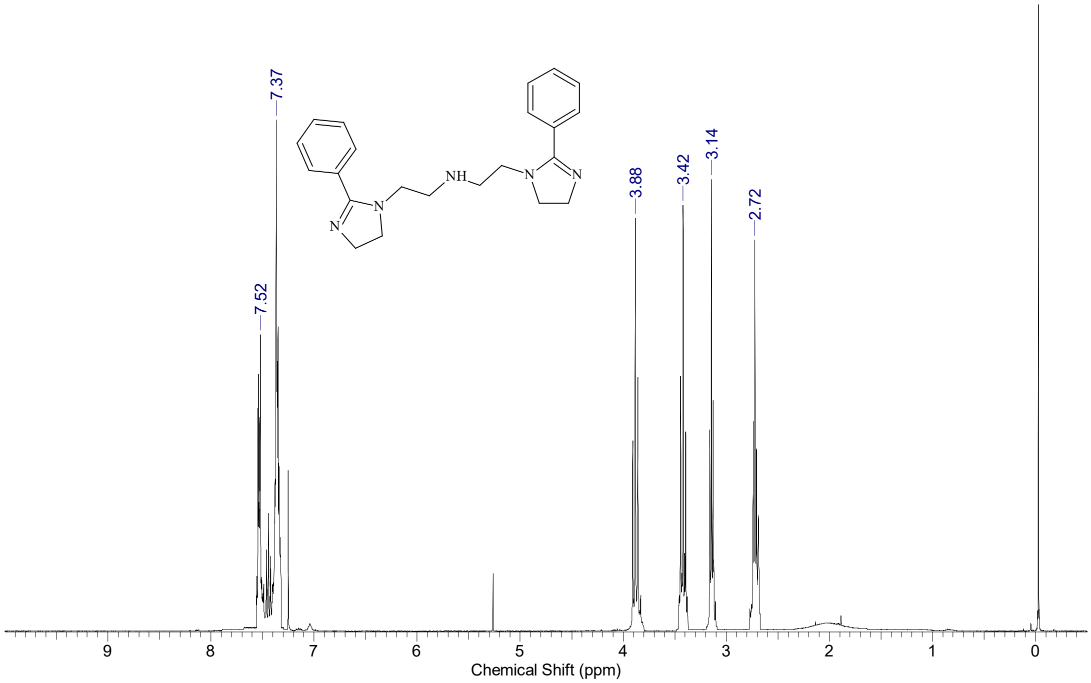

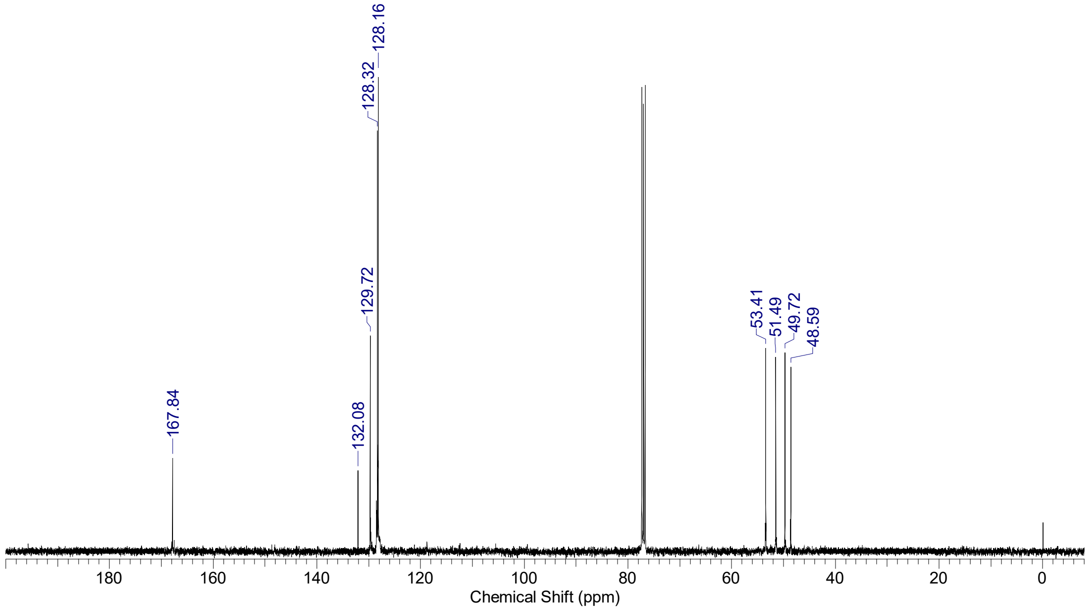

Fig. S6. The ^{1}H (400MHz) and ^{13}C (101MHz) NMR spectra for 3-(2-phenyl-4,5-dihydro-1H-imidazol-1-yl)propan-1-amine (1f) in $CDCl_3$

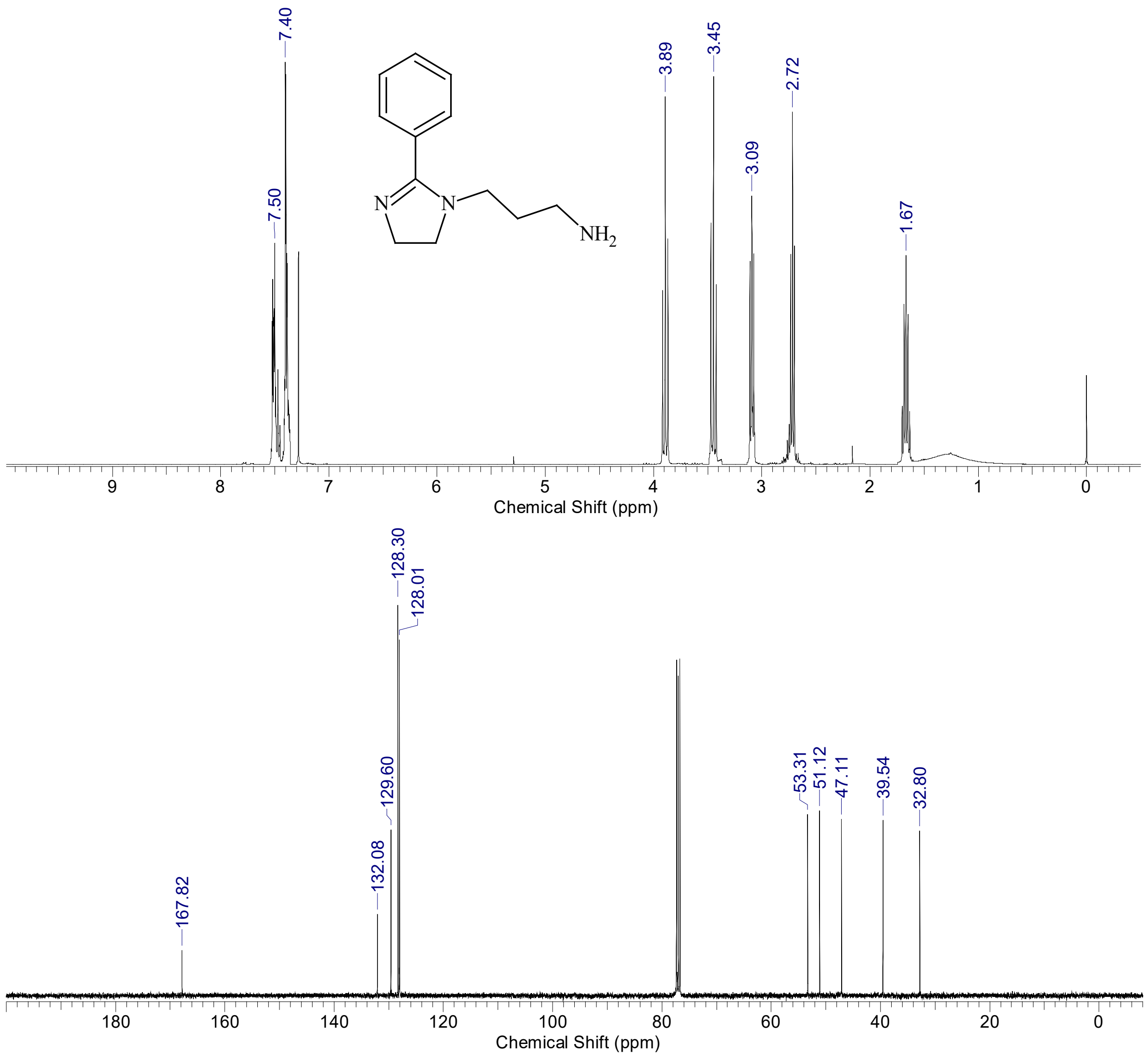

Fig. S7. The ^{1}H (400MHz) and ^{13}C (101MHz) NMR spectra for N-benzyl-1-(4,5-dihydro-1H-imidazol-2-yl)methanamine (1g) in $CDCl_3$

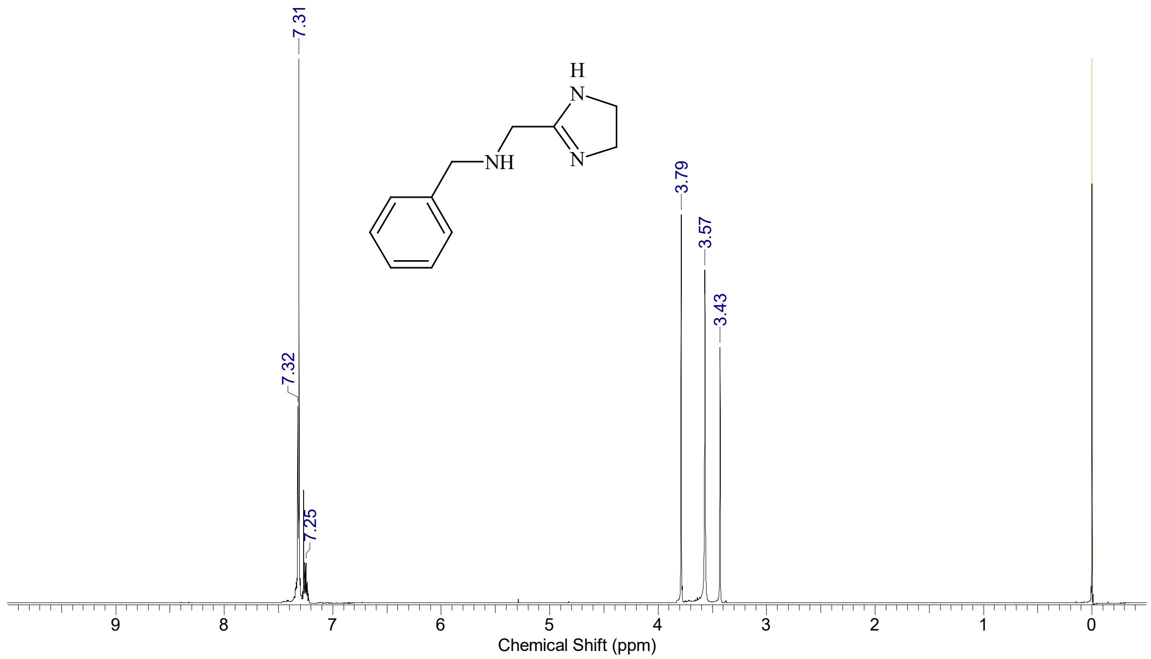

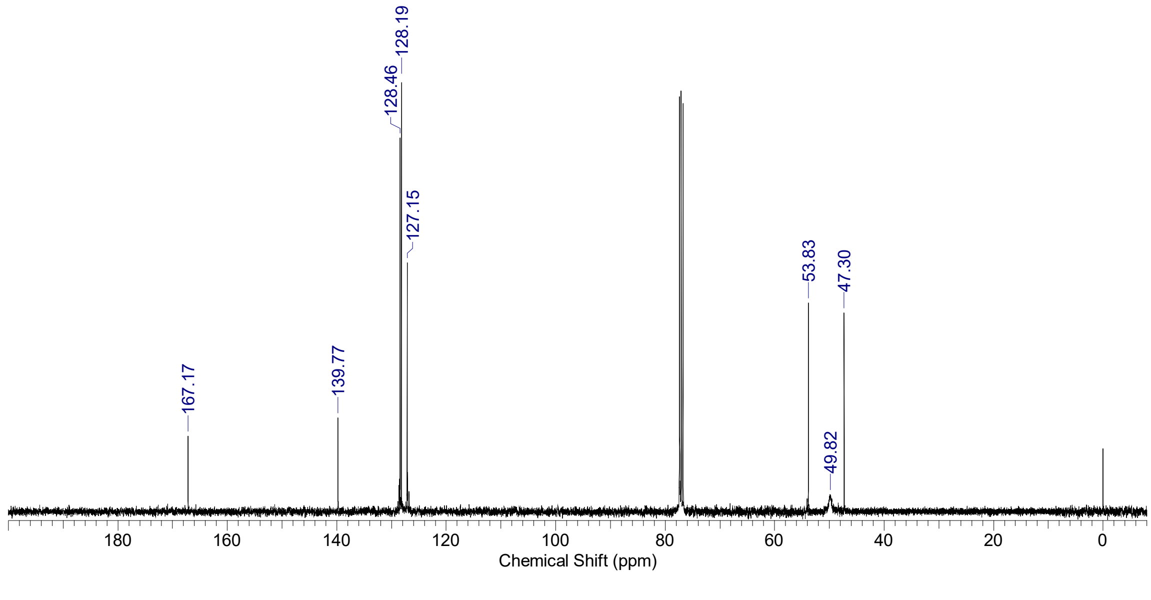

Fig. S8. The ^{1}H (400MHz) and ^{13}C (101MHz) NMR spectra for 2-{2-[(benzylamino)methyl]-4,5-dihydro-1H-imidazol-1-yl}ethanamine (1h) in $CDCl_3$

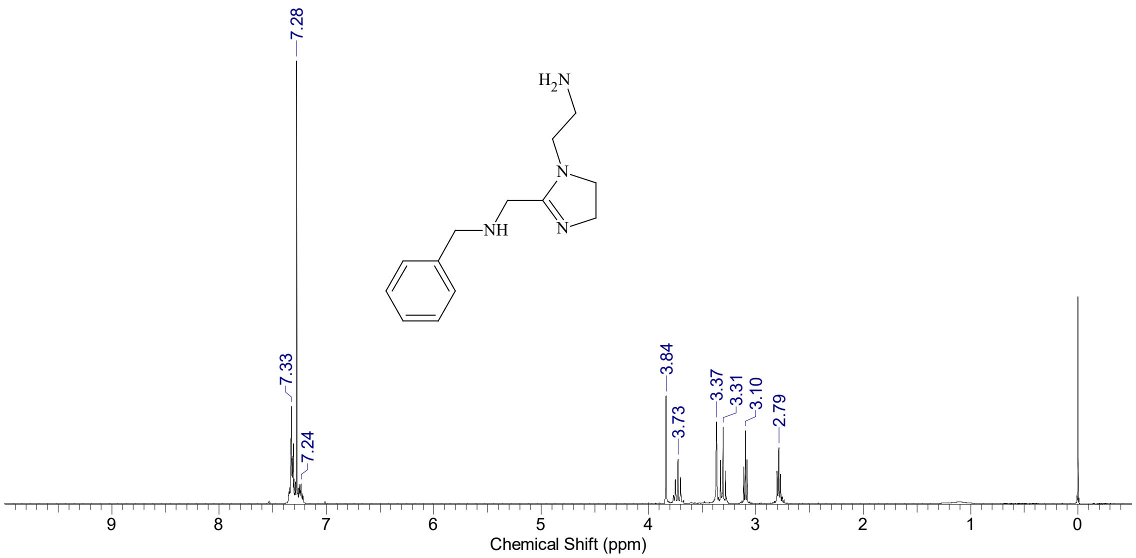

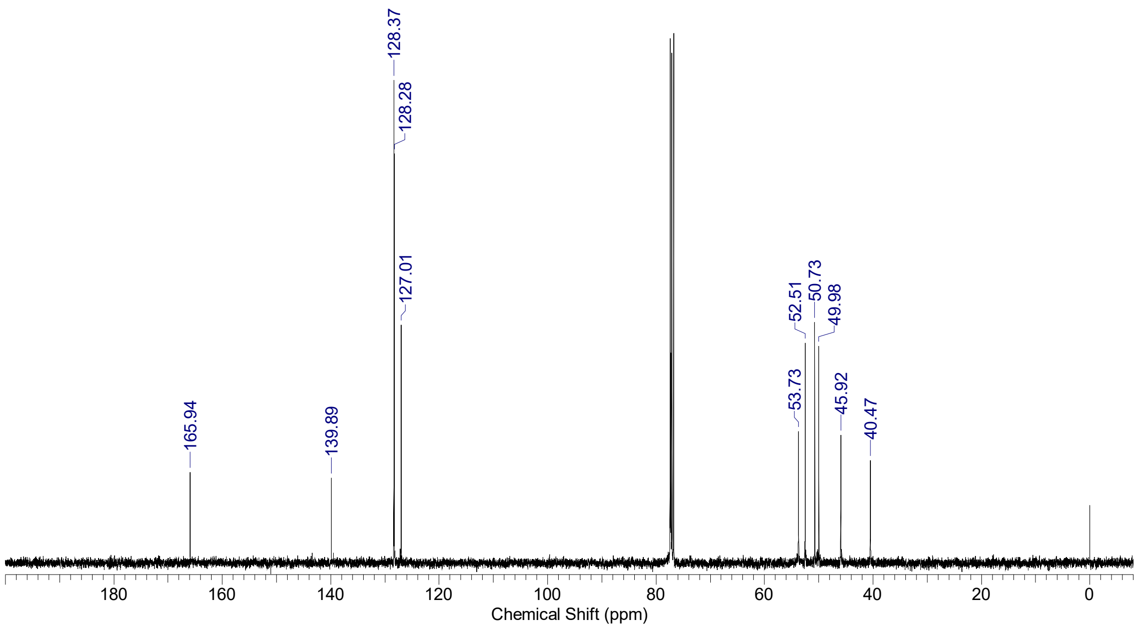

Fig. S9. The ^{1}H (400MHz) and ^{13}C (101MHz) NMR spectra for 2-phenyl-3a,4,5,6,7,7a-hexahydro-1H-benzimidazole (1i) in $CDCl_3$

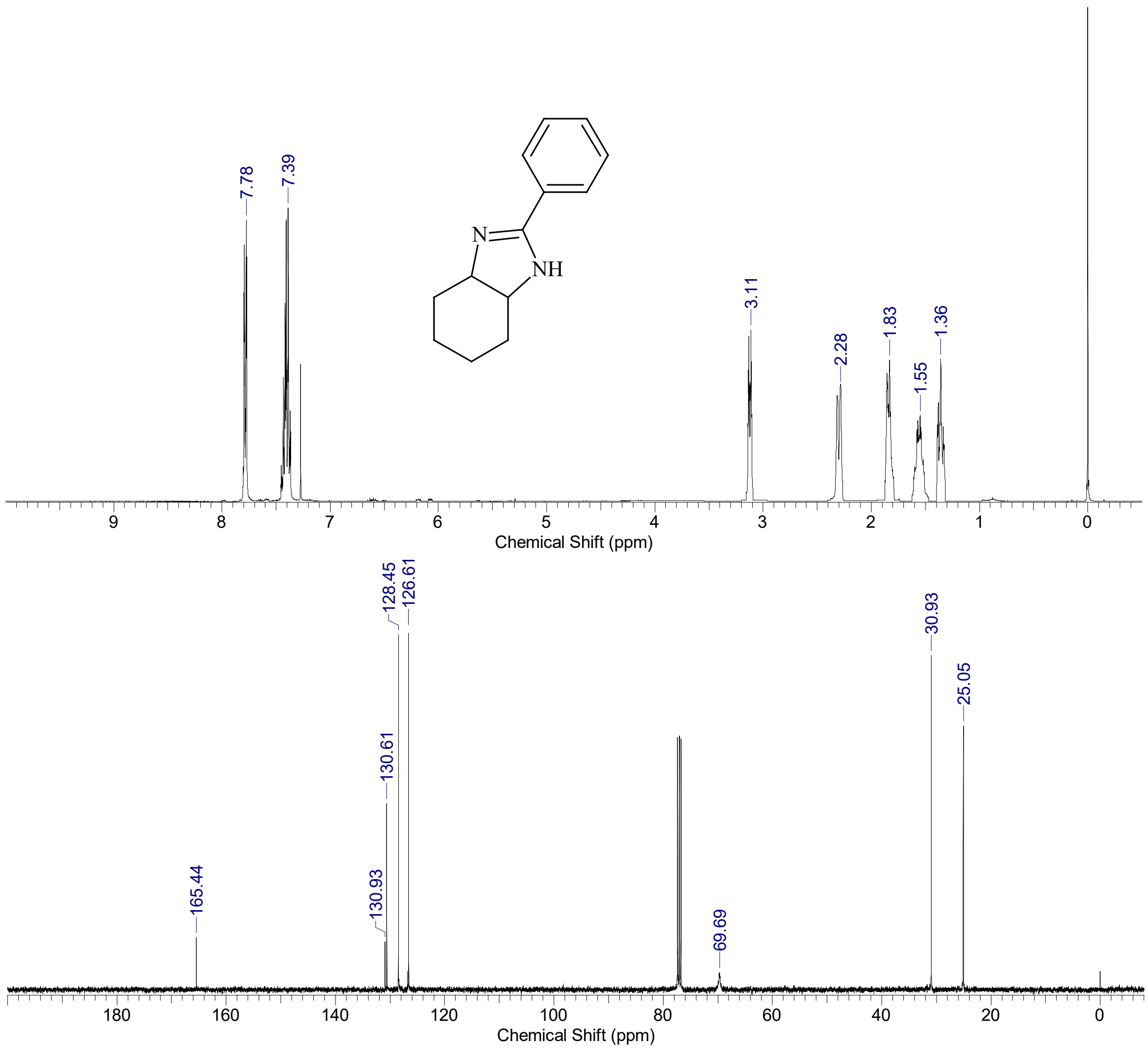

Fig. S10. The ^{1}H (400MHz) and ^{13}C (101MHz) NMR spectra for 2-phenyl-4,5-dihydro-1,3-oxazole (1j) in $CDCl_3$

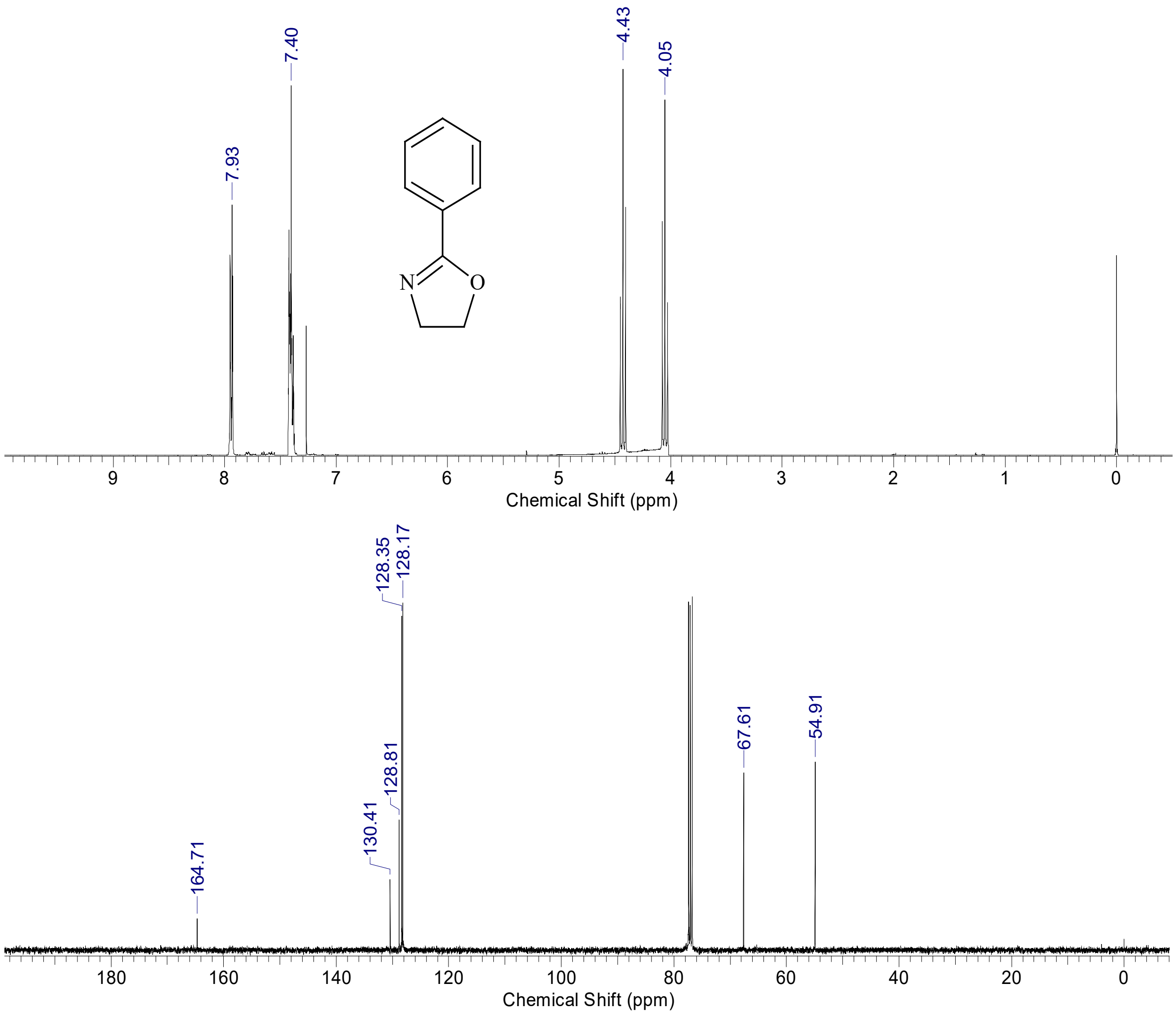

Fig. S11. The ^{1}H (400MHz) and ^{13}C (101MHz) NMR spectra for 3-(2-phenyl-5,6-dihydropyrimidin-1(4H)-yl)propan-1-amine (1k) in $CDCl_3$

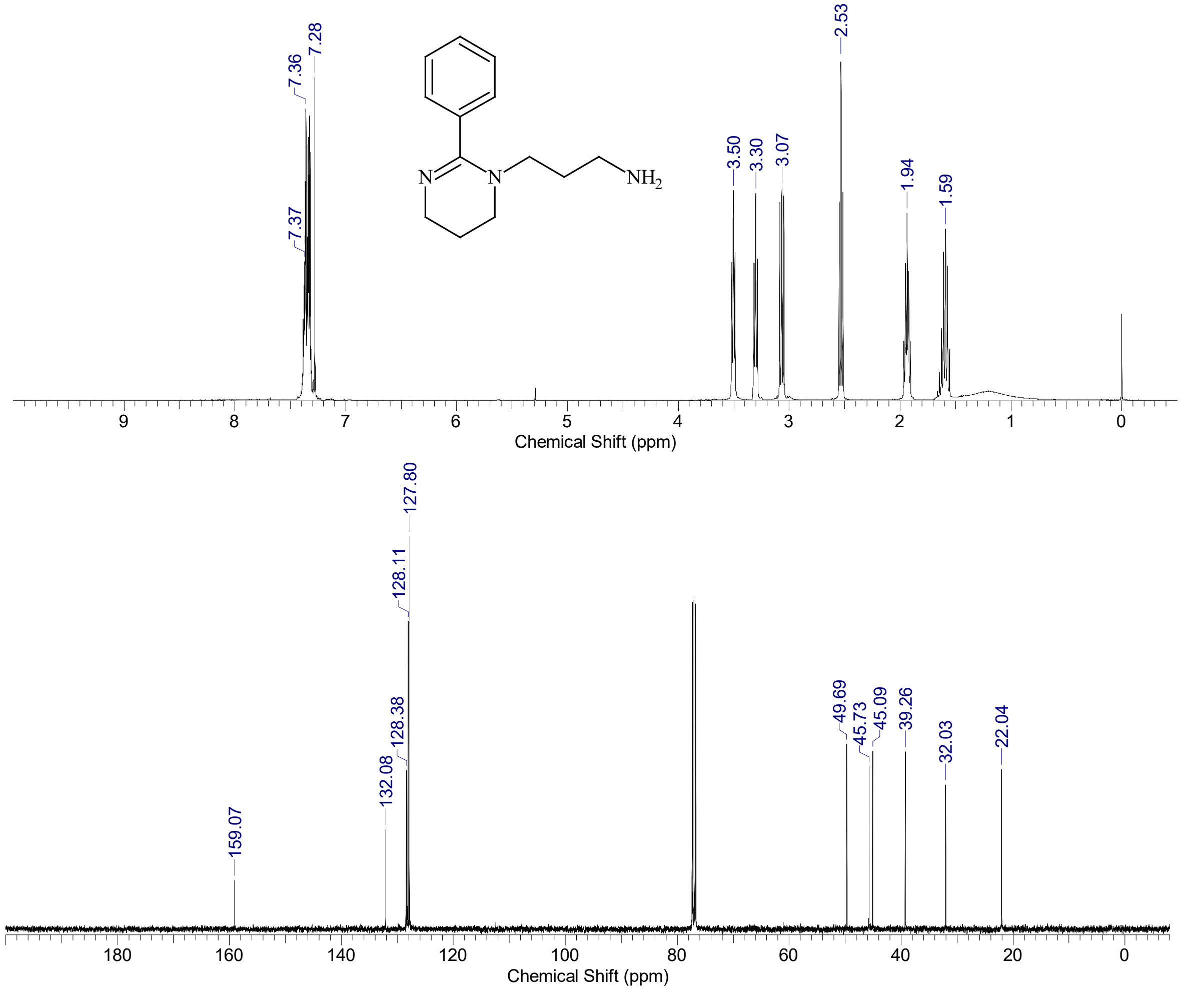

Fig. S12. The ^{1}H (400MHz) and ^{13}C (101MHz) NMR spectra for 1,1'-ethane-1,2-diylbis(2-phenyl-1,4,5,6-tetrahydropyrimidine) (1l) in $CDCl_3$

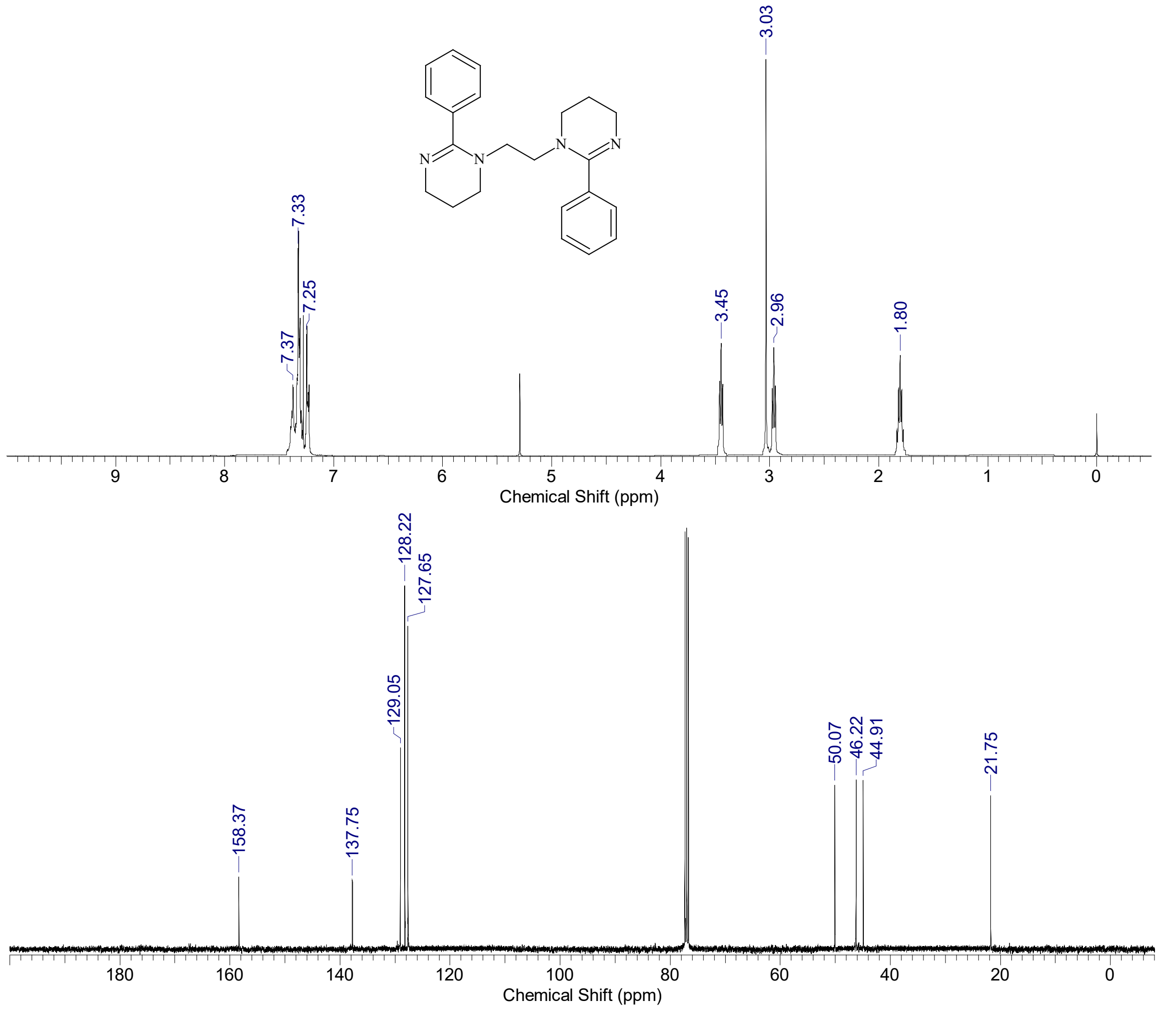

Fig. S13. The ^{1}H (400MHz) and ^{13}C (101MHz) NMR spectra for 2-phenyl-1,4,5,6-tetrahydropyrimidine (1m) in $CDCl_3$

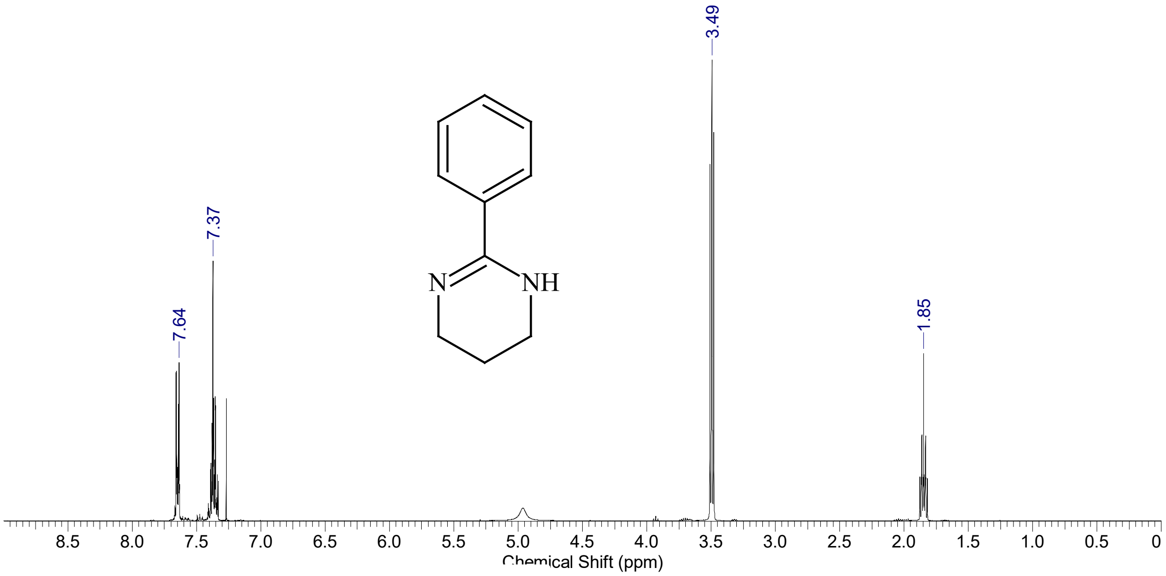

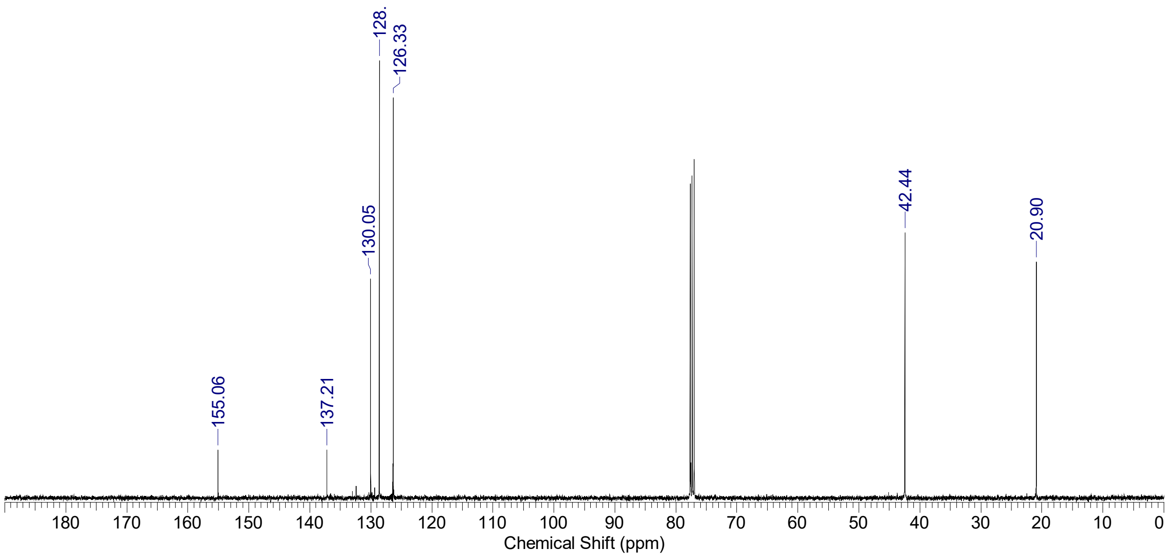

Fig. S14. The ^{1}H (400MHz) and ^{13}C (101MHz) NMR spectra for 2,2'-ethane-1,2-diyldi-1,4,5,6-tetrahydropyrimidine (1n) in $CDCl_3$

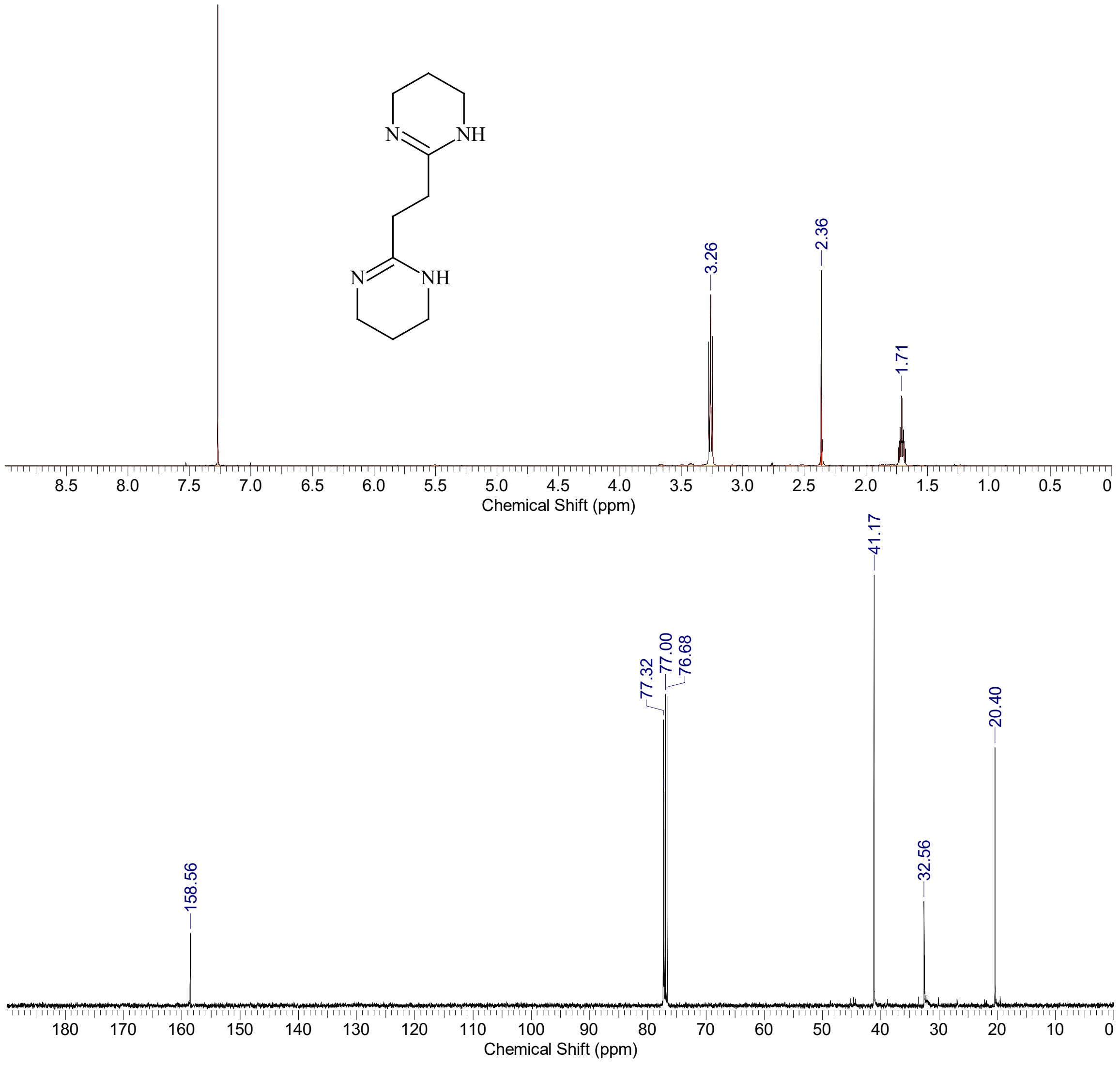

Fig. S15. The ^1H (400MHz) and ^{13}C (101MHz) NMR spectra for N-benzylethane-1,2-diamine (2a) in $CDCl_3$

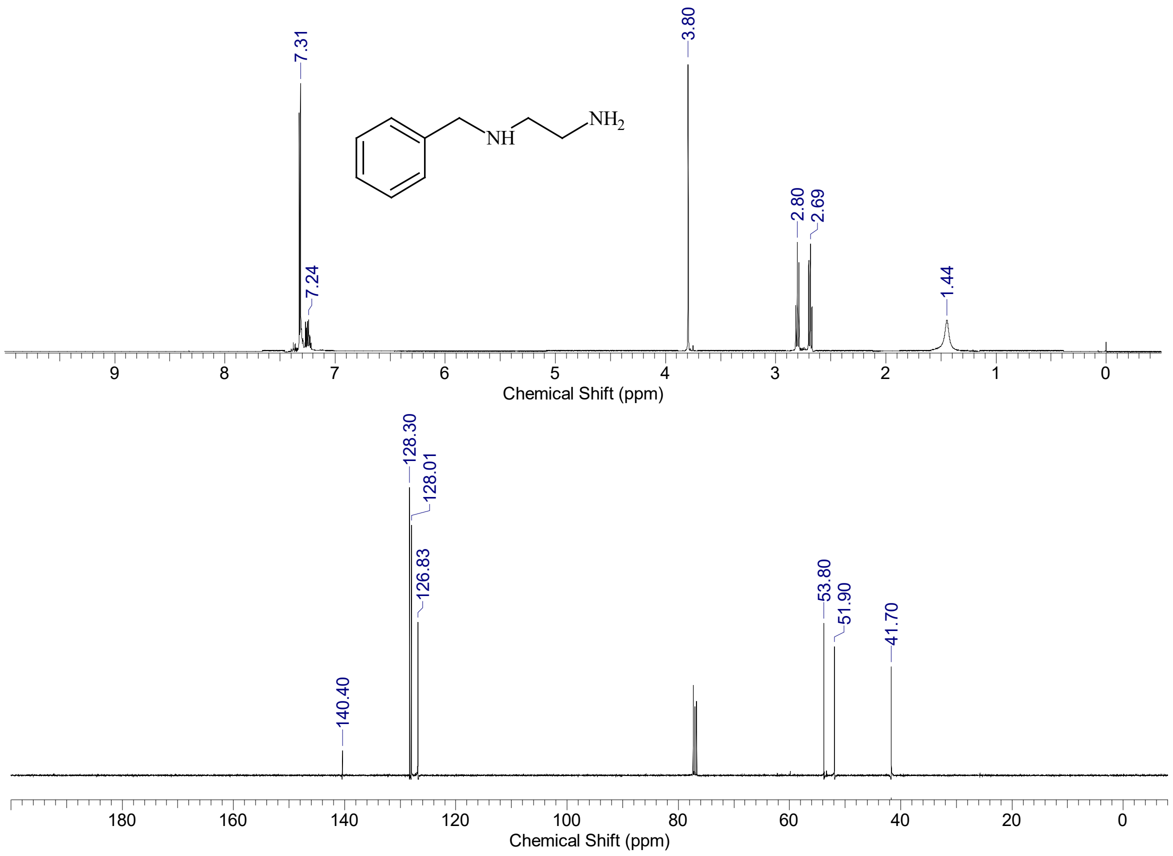

Fig. S16. The ^{1}H (400MHz) and ^{13}C (101MHz) NMR spectra for N-(2-aminoethyl)-N'-benzylethane-1,2-diamine (2b) in $CDCl_3$

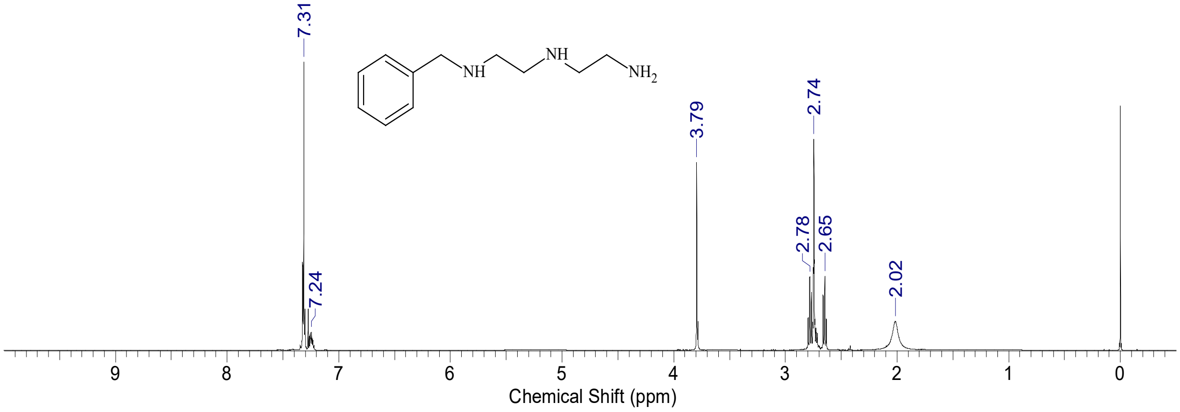

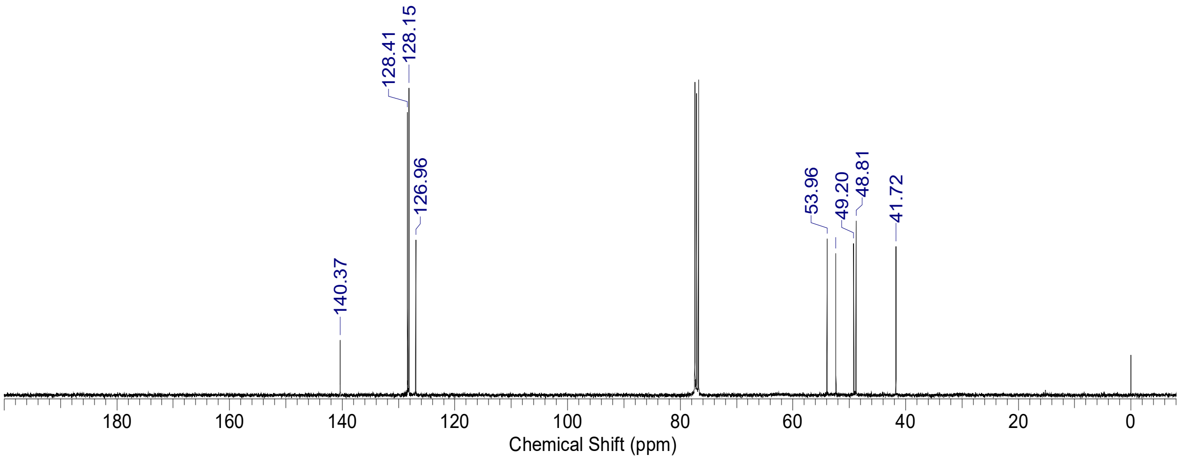

Fig. S17. The ^{1}H (400MHz) and ^{13}C (101MHz) NMR spectra for N-benzyl-N'-(2-{[2-(benzylamino)ethyl]amino}ethyl)ethane-1,2-diamine (2c) in $CDCl_3$

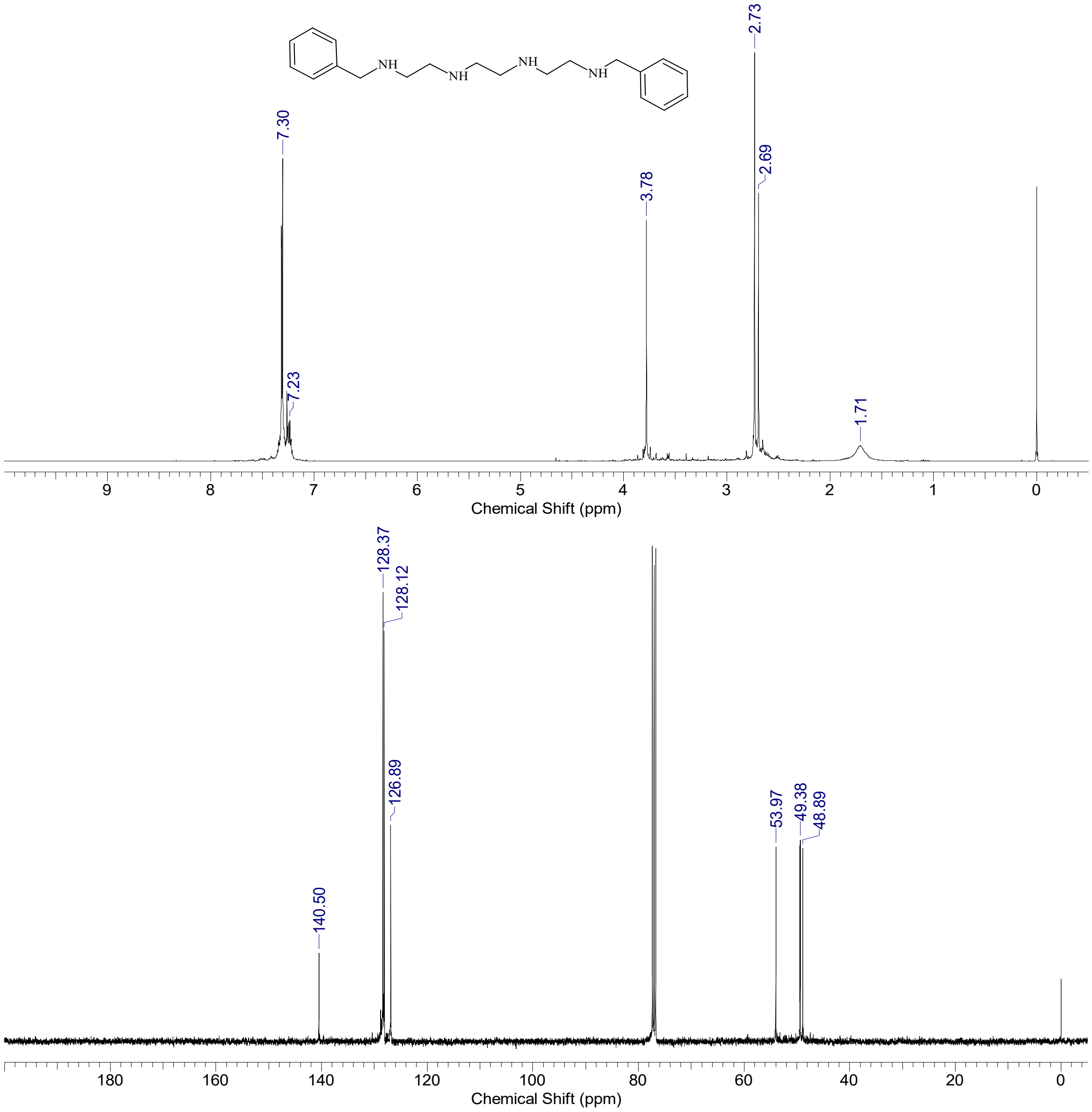

Fig. S18. The ^{1}H (400MHz) and ^{13}C (101MHz) NMR spectra for N-benzyl-N'-{2-[(2-{[2-(benzylamino)ethyl]amino}ethyl) amino]ethyl}ethane-1,2-diamine (2d) in $CDCl_3$

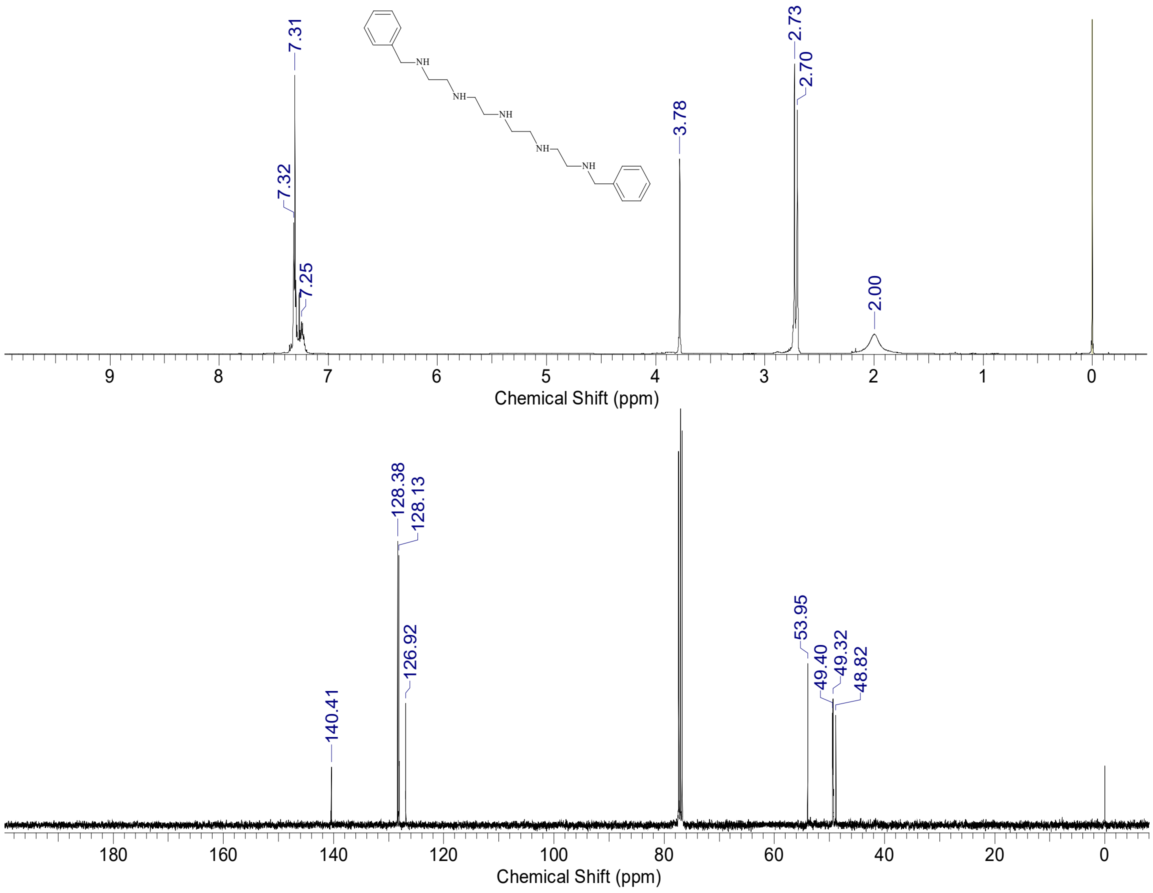

Fig. S19. The ^{1}H (400MHz) and ^{13}C (101MHz) NMR spectra for N-(2-aminoethyl)-N'-[2-(benzylamino)ethyl]ethane-1,2-diamine (2e) in $CDCl_3$

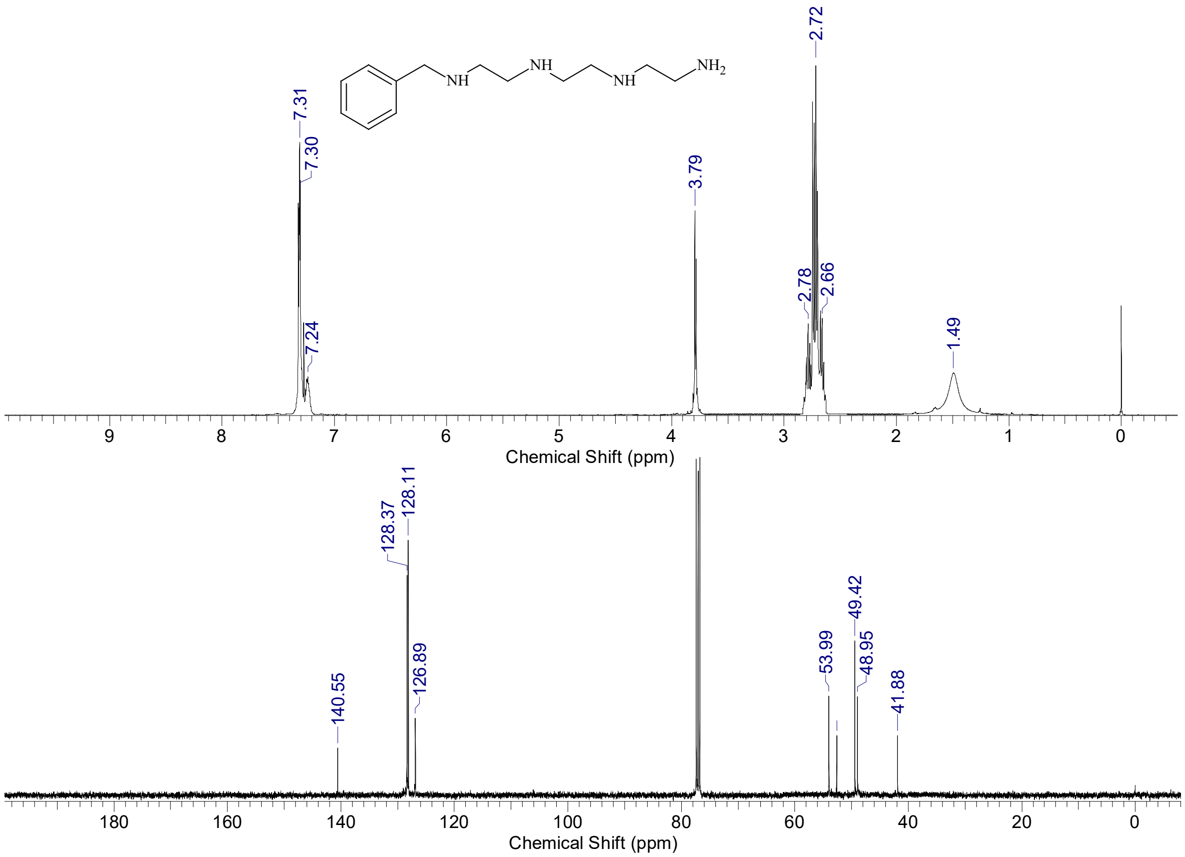

Fig. S20. The ^{1}H (400MHz) and ^{13}C (101MHz) NMR spectra for 2-(benzylamino)ethanol (2f) in $CDCl_3$

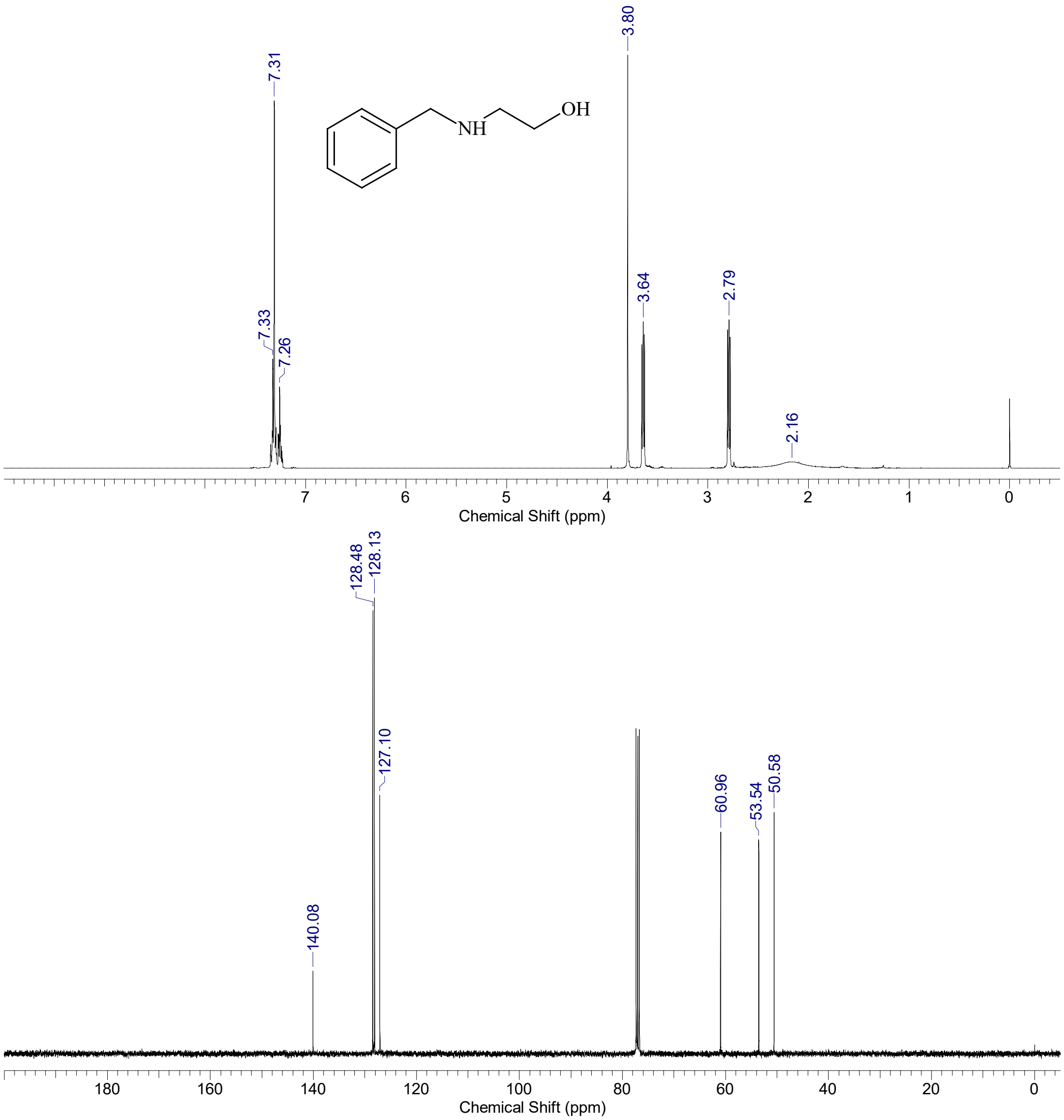

Fig. S21. The ^{1}H (400MHz) and ^{13}C (101MHz) NMR spectra for N-(3-aminopropyl)-N'-benzylpropane-1,3-diamine (2g) in $CDCl_3$

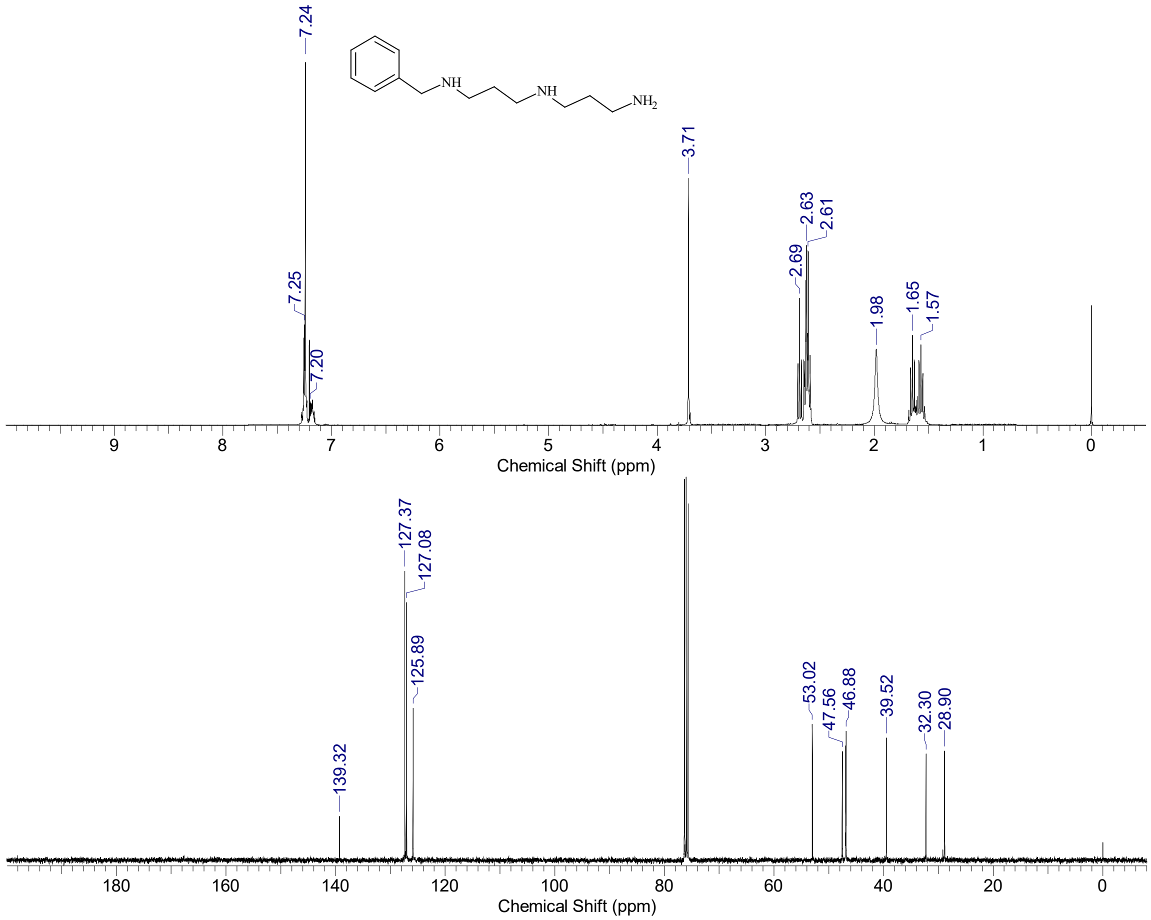

Fig. S22. The ^{1}H (400MHz) and ^{13}C (101MHz) NMR spectra for N-benzylpropane-1,3-diamine (2h) in $CDCl_3$

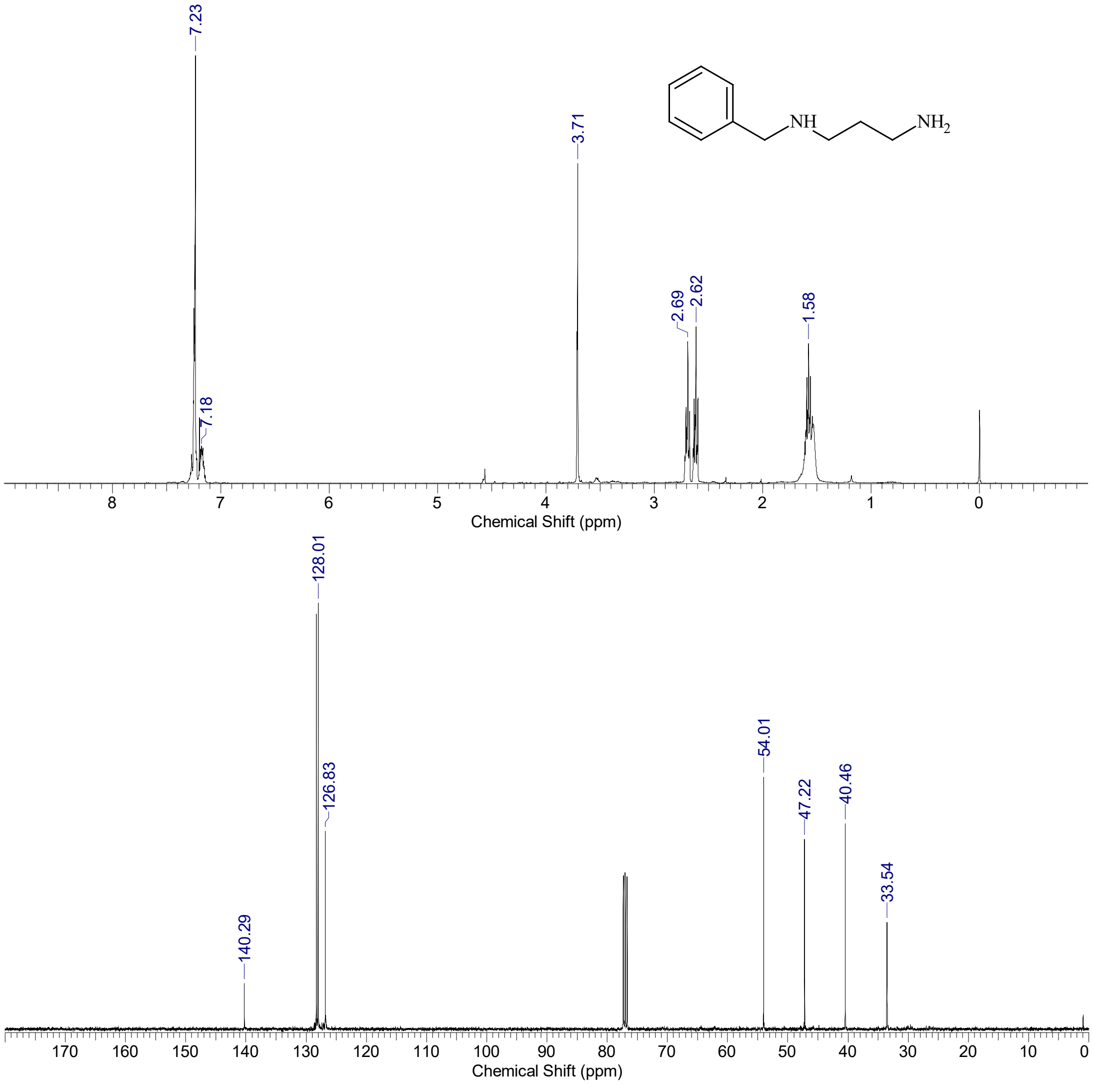

Fig. S23. The ^{1}H (400MHz) and ^{13}C (101MHz) NMR spectra for Spermidine (2i) in $CDCl_3$

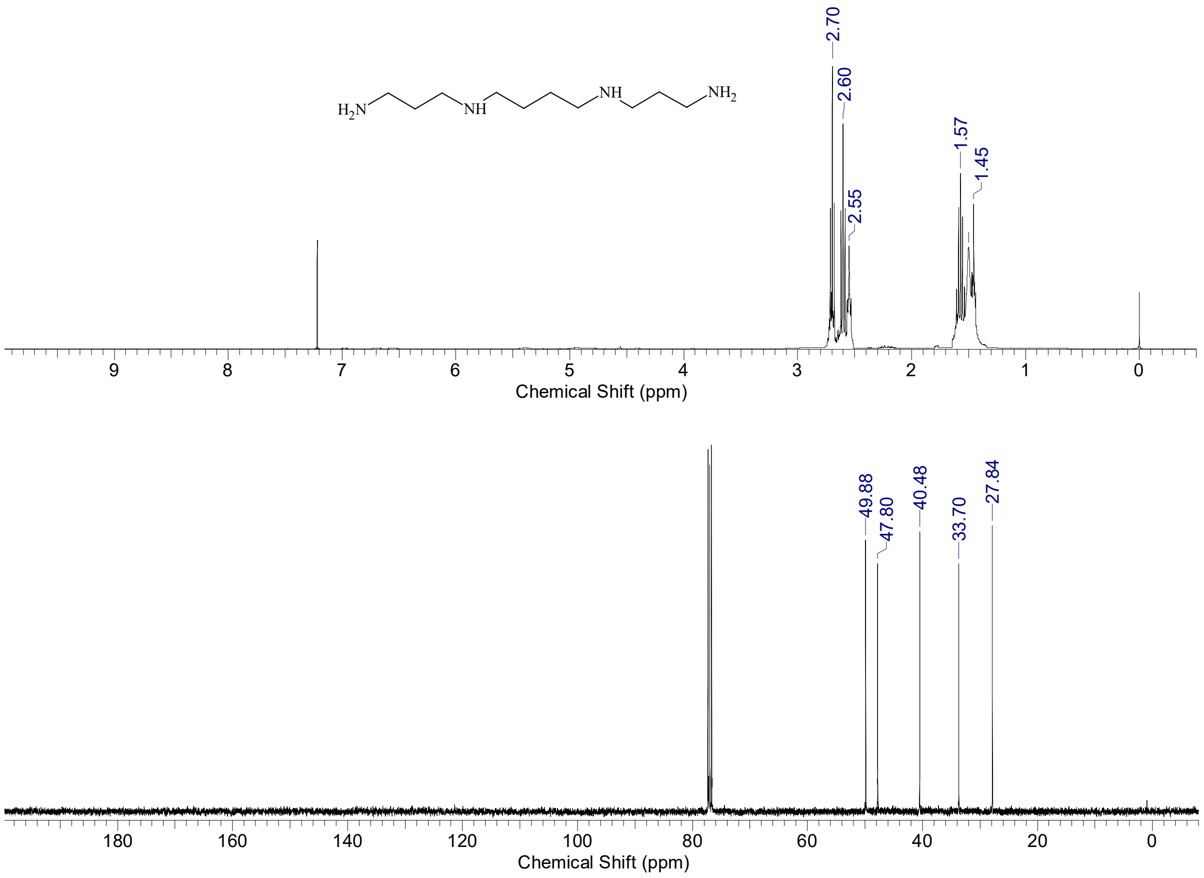

Fig. S24. The ^{1}H (400MHz) and ^{13}C (101MHz) NMR spectra for N-(2-aminoethyl) benzamide (3) in $CDCl_3$

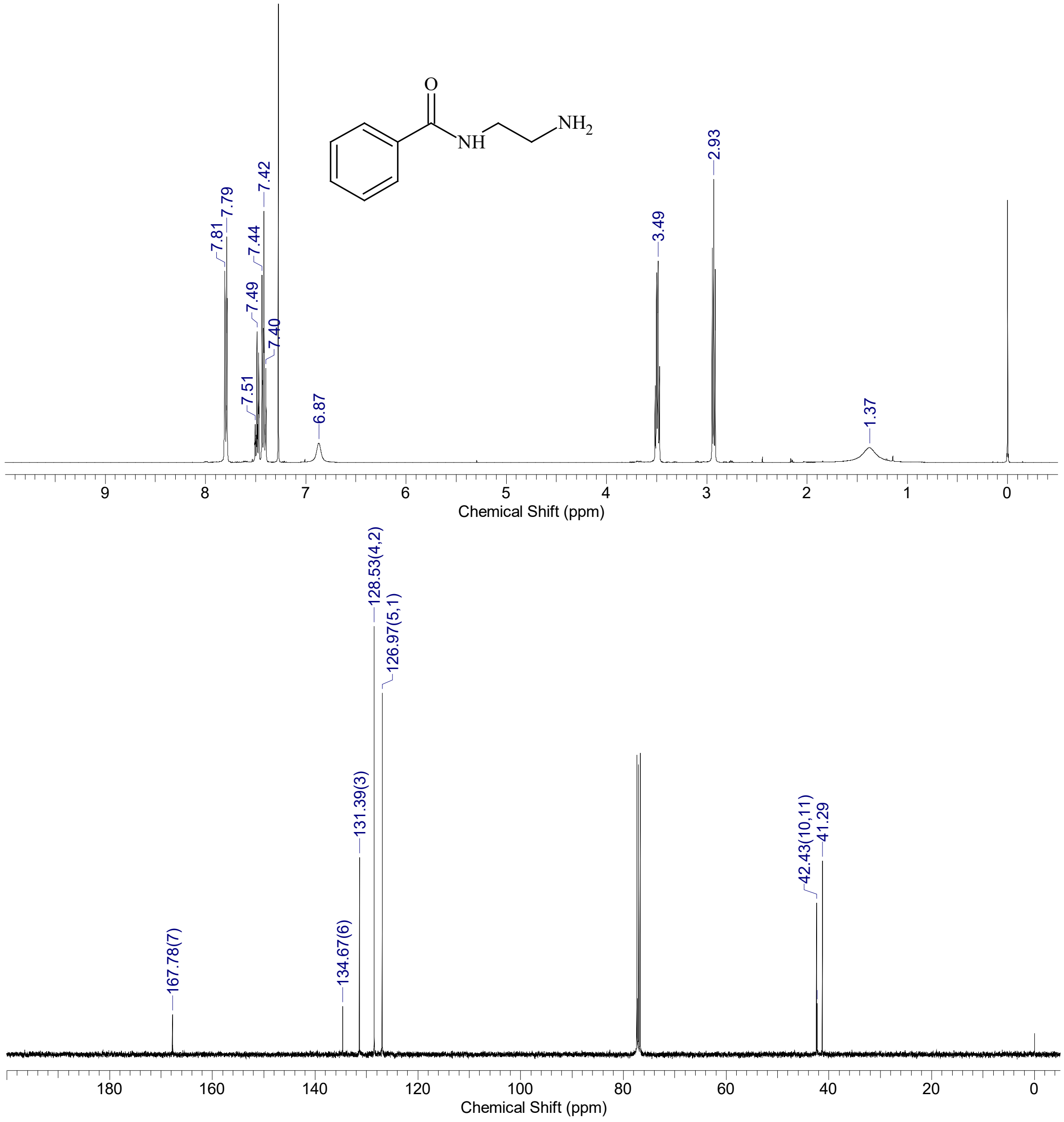

Fig. S25. The ^{1}H (400MHz) and ^{13}C (101MHz) NMR spectra for N-{2-[(cyanomethyl)amino]ethyl}benzamide (4) in $CDCl_3$

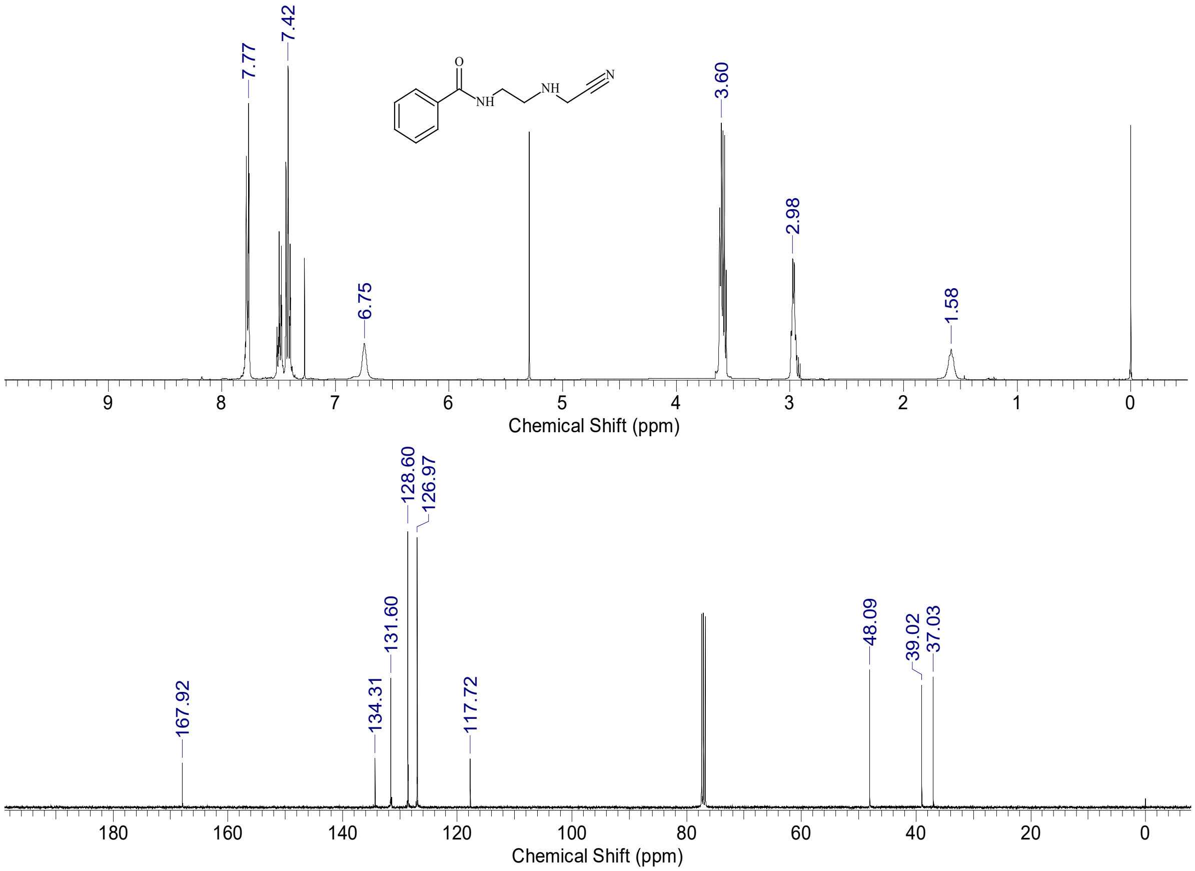

Fig. S26. The ^{1}H (400MHz) and ^{13}C (101MHz) NMR spectra for N-{2-[bis(cyanomethyl)amino]ethyl}benzamide (5) in $CDCl_3$

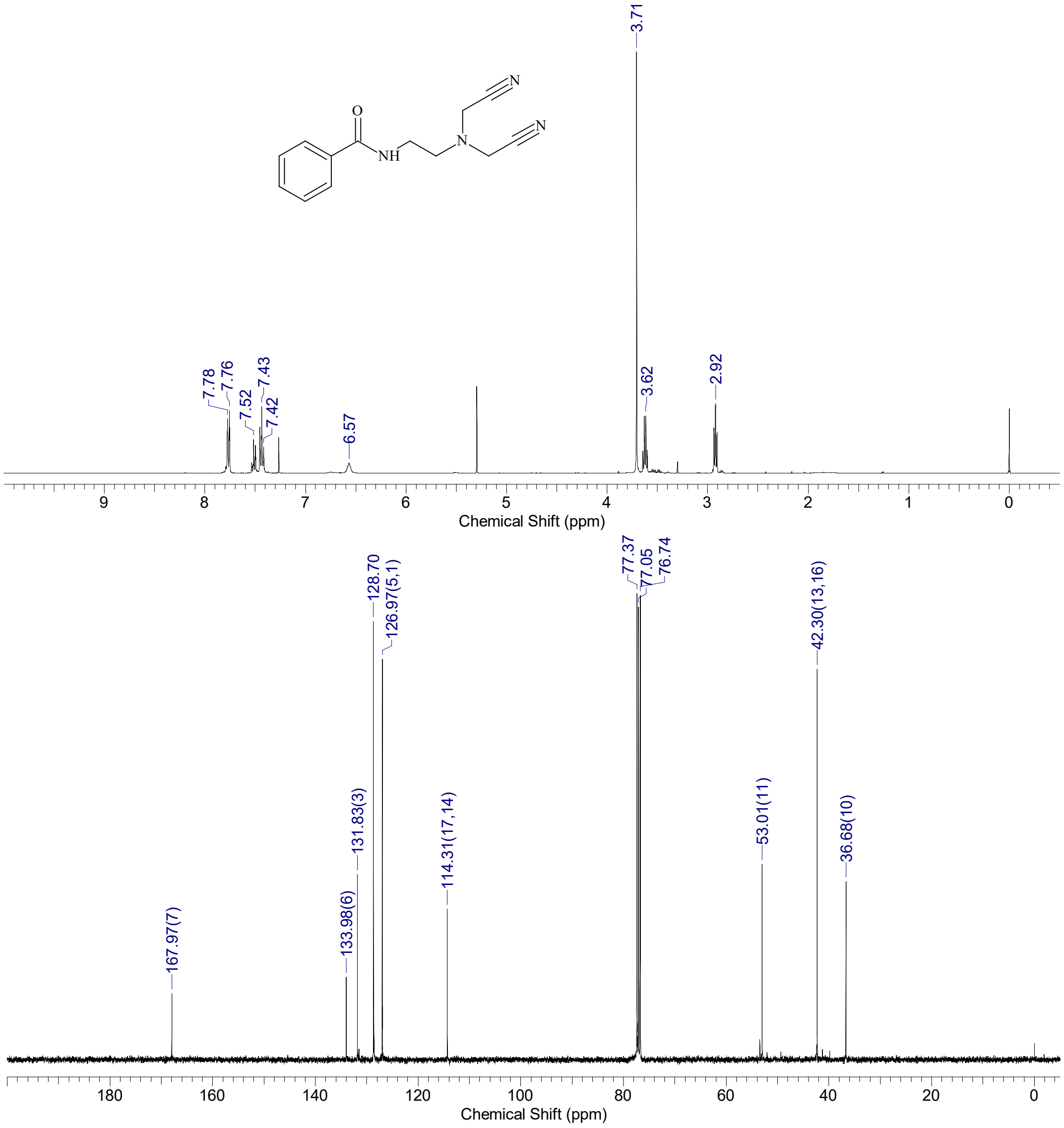

Fig. S27. The ^{1}H (400MHz) and ^{13}C (101MHz) NMR spectra for N-{2-[bis(4,5-dihydro-1H-imidazol-2-ylmethyl)amino]ethyl} benzamide (6) in $CDCl_3$

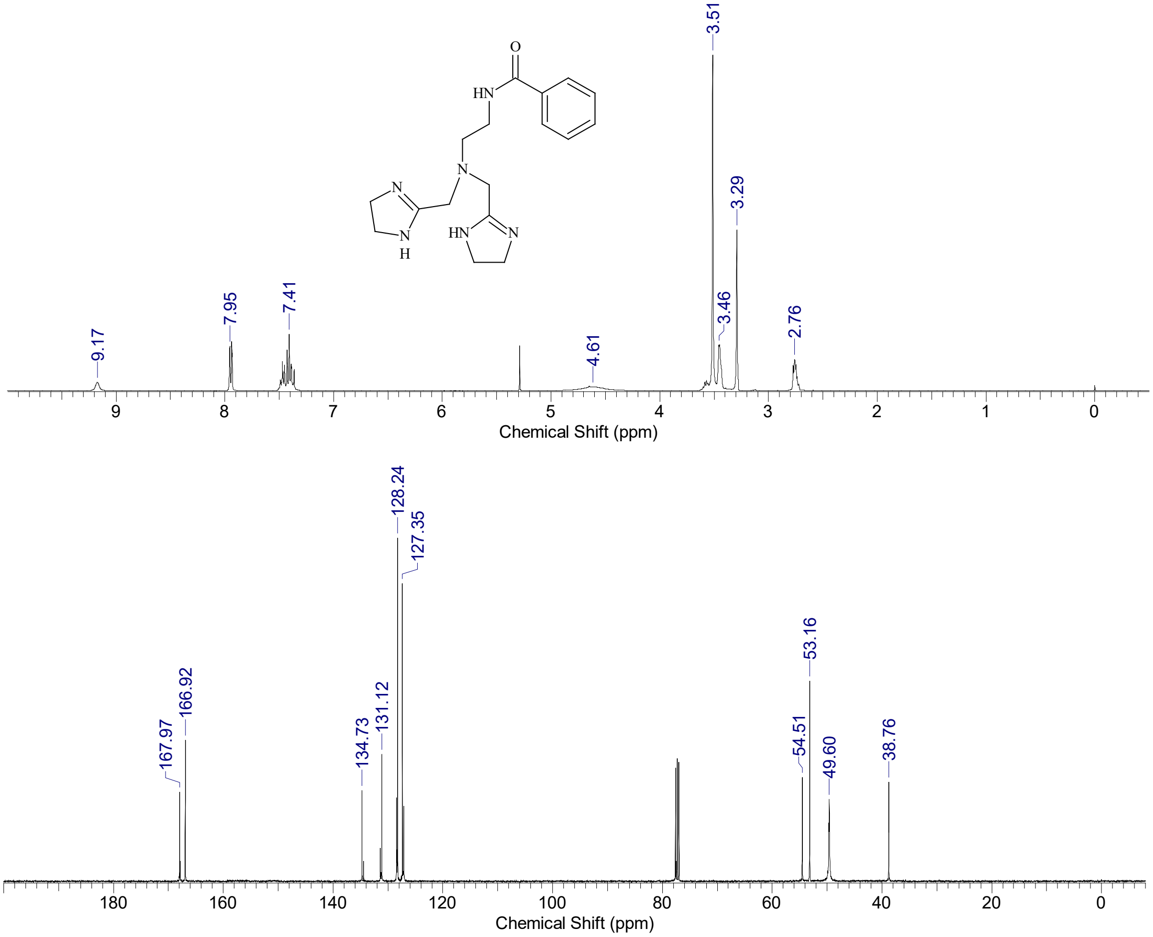

Fig. S28. The ^{1}H (400MHz) and ^{13}C (101MHz) NMR spectra for N'-(2-aminoethyl)-N-{2-[(2-aminoethyl)amino]ethyl}-N-[2-(benzylamino)ethyl]ethane-1,2-diamine (7) in $CDCl_3$

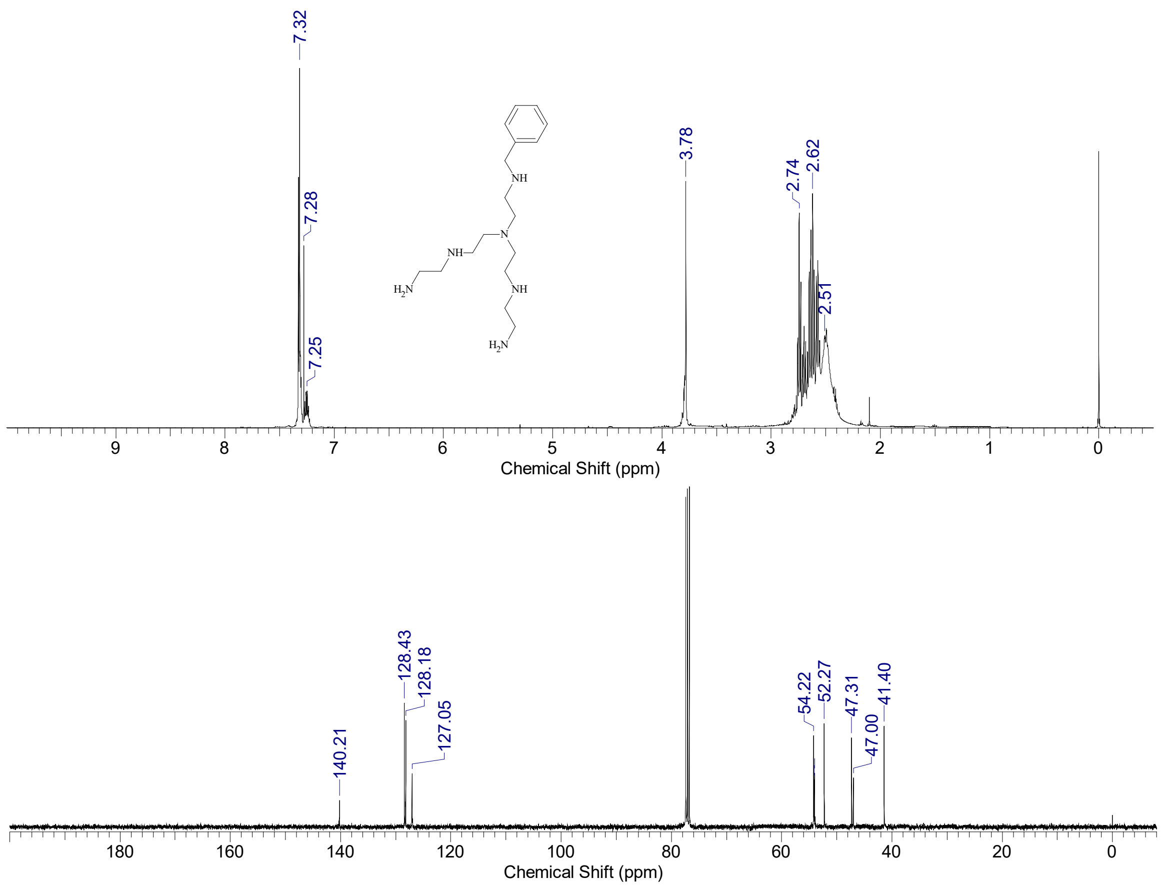

Fig. S29. **The ^{1}H (400MHz) and ^{13}C (101MHz) NMR spectra for di-tert-butyl (((((2-(benzylamino)ethyl)azanediyl)bis(ethane-2,1-diyl))bis(azanediyl))bis(ethane-2,1-diyl))dicarbamate (7a) in $CDCl_3$**

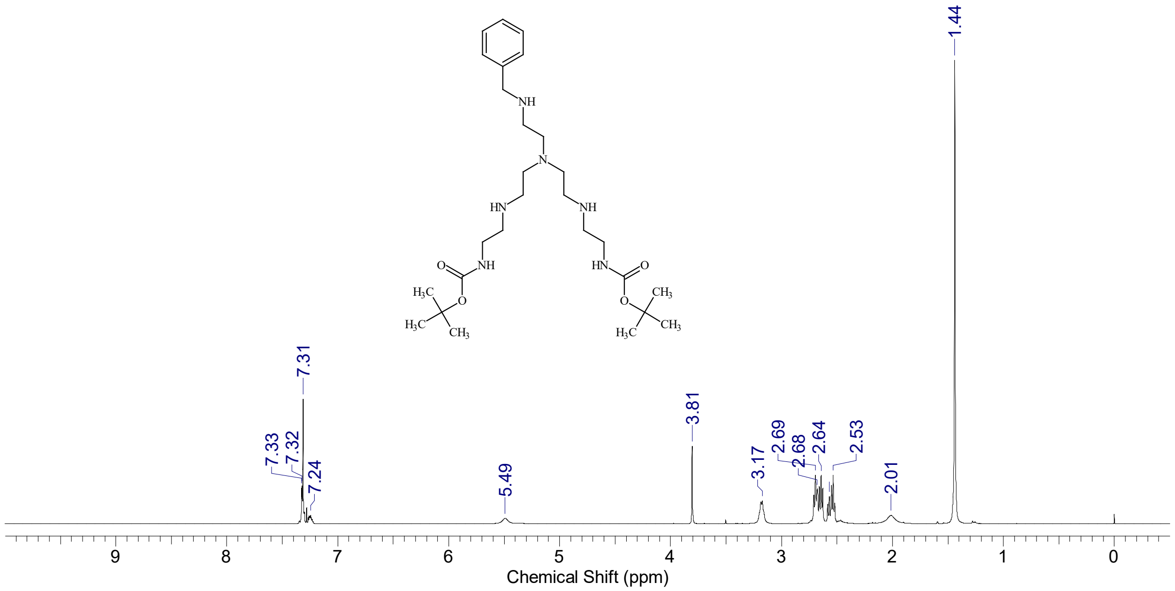

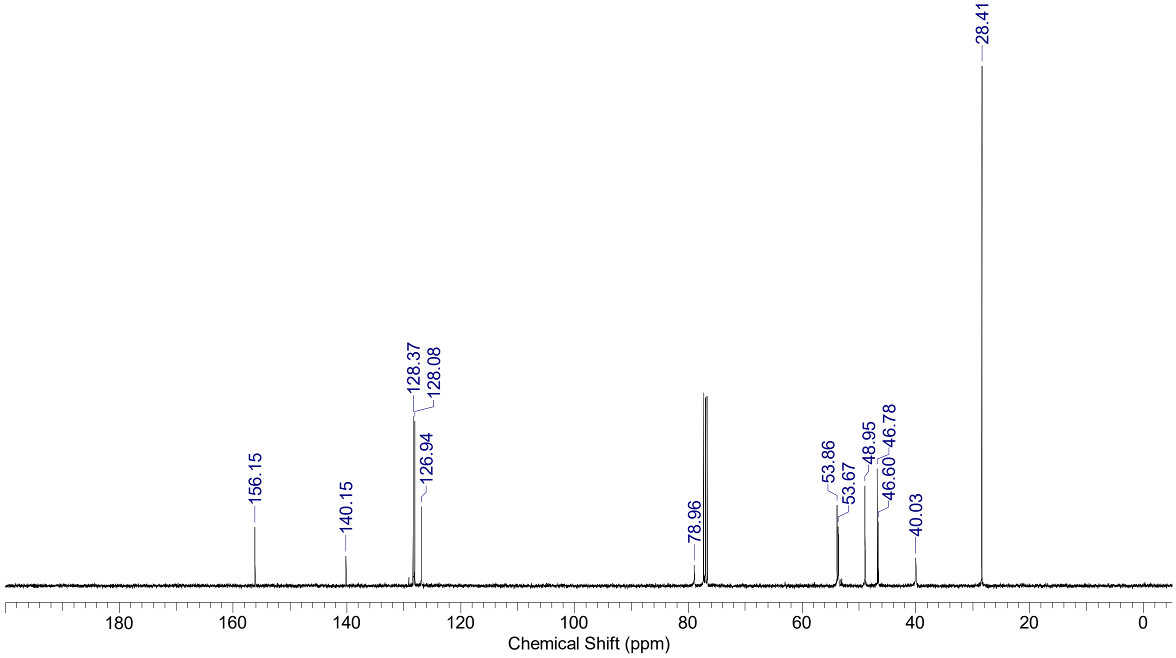

Fig. S30. The ^{1}H (400MHz) and ^{13}C (101MHz) NMR spectra for di-tert-butyl (((((2-aminoethyl)azanediyl)bis(ethane-2,1-diyl))bis(azanediyl))bis(ethane-2,1-diyl))dicarbamate (7b) in $CDCl_3$

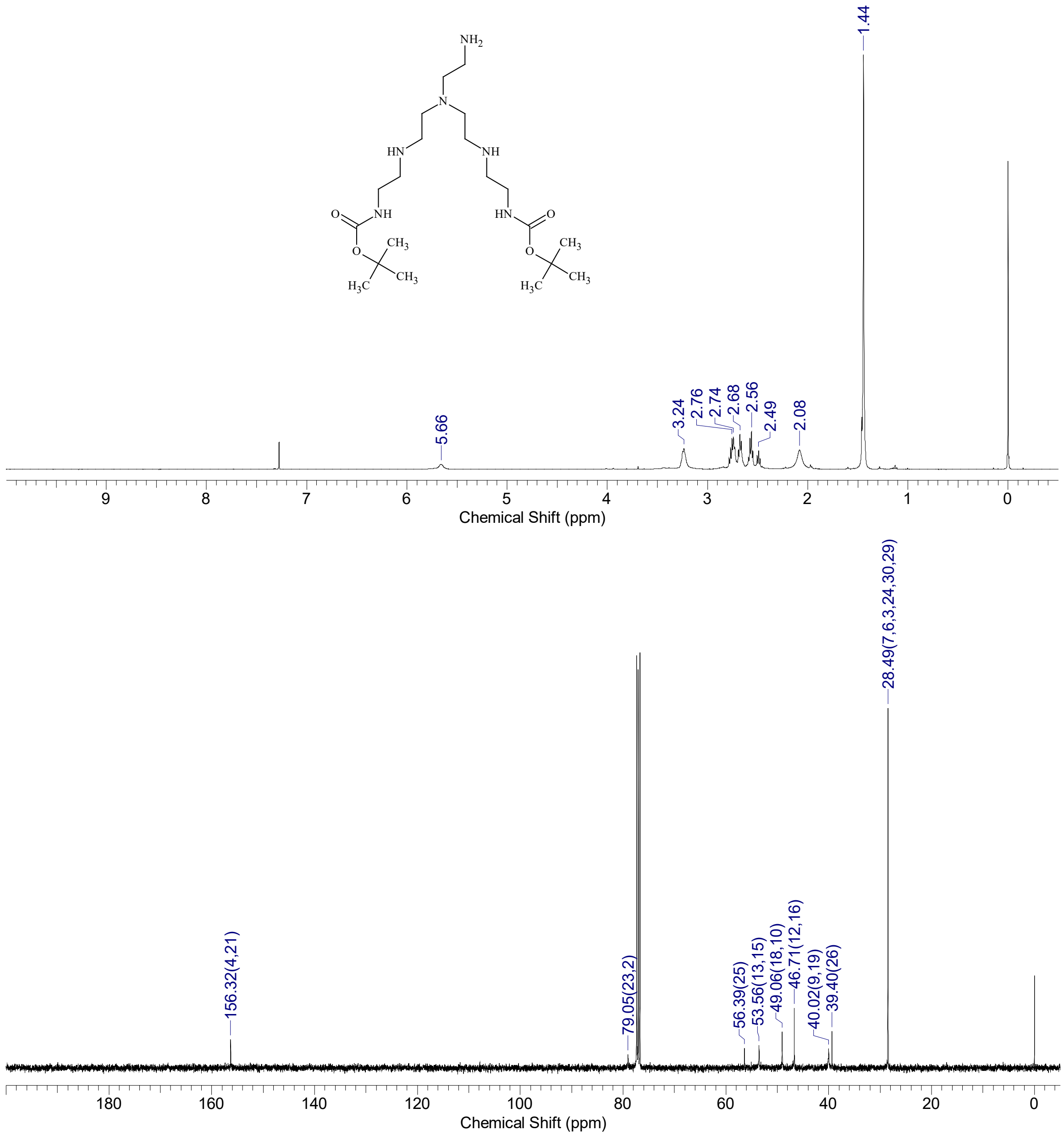

Fig. S31. The ^{1}H (400MHz) and ^{13}C (101MHz) NMR spectra for di-tert-butyl (((((2-((4-amino-1-methyl-5-nitroso-6-oxo-1,6-dihydropyrimidin-2-yl)amino)ethyl)azanediyl)bis(ethane-2,1-diyl))bis(azanediyl))bis(ethane-2,1-diyl))dicarbamate (8) in DMSO-d6

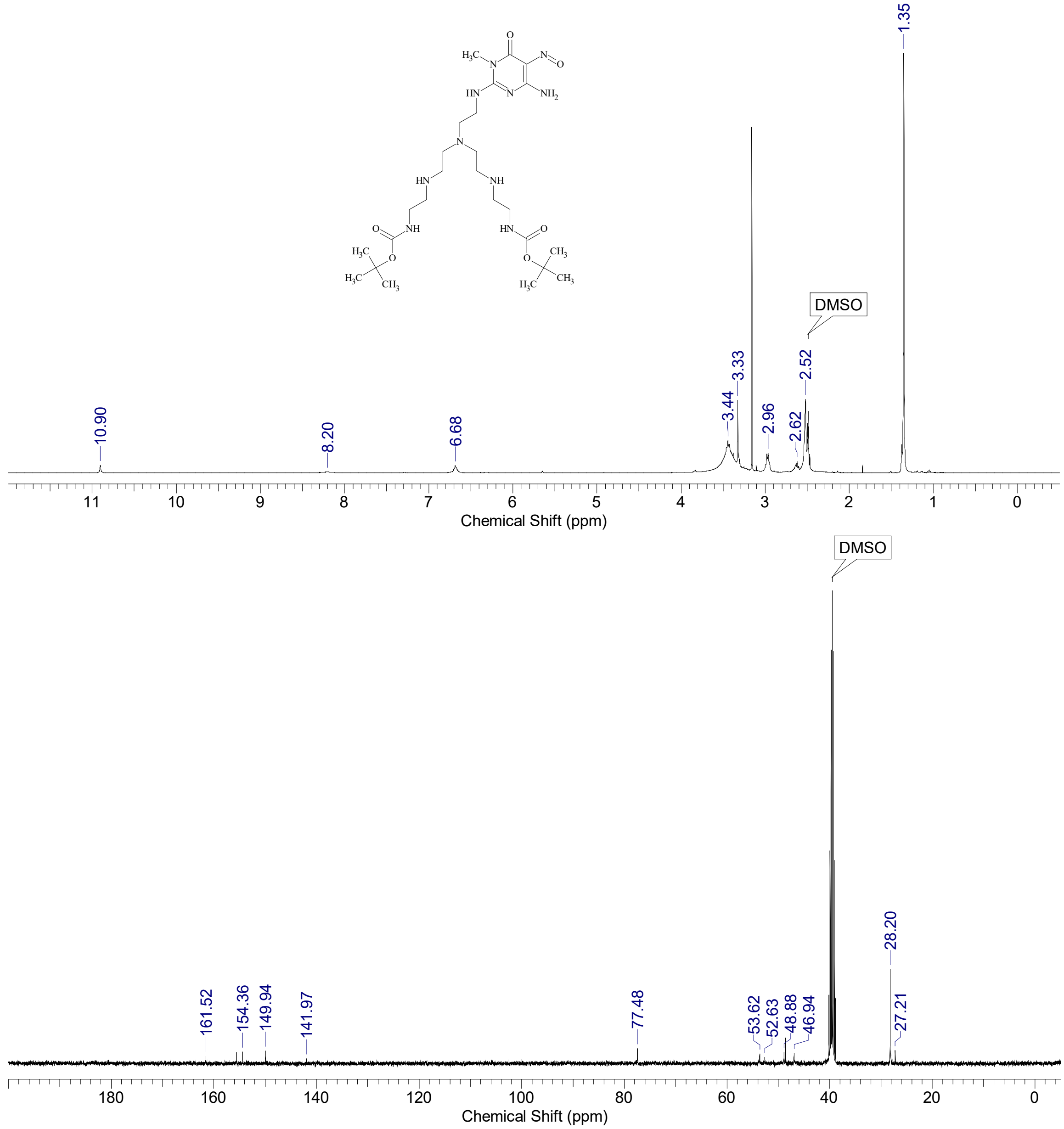

Supplement: Supplementary file 1 — jo3c02128_si_001.pdf [file jo3c02128_si_001.pdf]
